# Dietary Astragalus and Fermented Astragalus in Postpartum Turpan Black Ewes: Impacts on Maternal Health, Rumen Microbiota, Lactation, and Lamb Development

**DOI:** 10.3390/microorganisms14081701

**Published:** 2026-08-03

**Authors:** Hao Lu, Hui Chen, Tingting Li, Tingting Lu, Reyim Reyilaguli, Haibo Lv, Xihu Wang, Jianjun Zhang, Shijie Li, Xiaojun Liu, Jinping Ma, Rui Xiao, Guodong Zhao

**Affiliations:** 1 Xinjiang Herbivore Nutrition Laboratory for Meat & Milk, College of Animal Science, Xinjiang Agricultural University, Urumqi 830052, China; 15569593918@163.com (H.L.); m13201308339@163.com (H.C.); litingting_2025@163.com (T.L.); lutt11071024@163.com (T.L.); 2 Huishang Ecological Animal Husbandry Co., Ltd., Turpan 838100, China; reylagul91@yeah.net (R.R.); 17799707298@163.com (H.L.); 3 Xinjiang Hutubi Breeding Cattle Farm Co., Ltd., Changji 831203, China; 18935737229@163.com (X.W.); zjj610227756@163.com (J.Z.); 18997546079@163.com (S.L.); 15999100356@163.com (X.L.); xmgs015@163.com (J.M.); 4 Xinjiang Animal Embryo Engineering Technology Research Center, Changji 831203, China; 13999363873@163.com

**Keywords:** astragalus, fermented astragalus, immune performance, lambs, postpartum ewes

## Abstract

Raw and fermented Astragalus membranaceus (AM) serve promising herbal additives for small ruminants, yet their integrated regulatory functions across lactating ewes and suckling lambs are insufficiently characterized. This study aimed to elucidate how dietary supplementation with raw and fermented Astragalus membranaceus modulates ruminal microbial communities, circulating metabolites, redox homeostasis, and lactation performance in postpartum Turpan black ewes, and to characterize the consequent effects on immune development and body weight gain in their offspring. The findings are intended to inform evidence-based strategies for herbal supplementation in sheep production. Forty-five postpartum Turpan black ewes were randomly allocated to three groups (*n* = 15): control (basal diet), AM and FAM groups. Ewe plasma, rumen fluid, milk yield, milk composition and serum antioxidant indices were sampled periodically. Lamb body weight, plasma antioxidants, immunoglobulins, growth hormone (GH) and insulin-like growth factor-1 (IGF-1) were measured every 15 days. Rumen 16S rRNA sequencing and untargeted plasma metabolomics were performed on day 45 post-partum. The group of FAM significantly increased milk yield (*p* < 0.05) and markedly improved total antioxidant capacity in ewes (*p* < 0.01). In the AM group, metabolites such as linoleic acid were significantly downregulated (*p* < 0.05), with enrichment in the linoleic acid metabolism pathway, while flavonoid metabolites, including quercetin 4′-isobutyrate, were strongly upregulated (*p* < 0.01). In the FAM group, tetradecanoic acid, palmitoleic acid, and related metabolites were significantly reduced (*p* < 0.05), and these differential metabolites showed significant enrichment in fatty acid biosynthesis (*p* < 0.05). Flavonoid compounds represented by melilotocarpan E were also significantly upregulated (*p* < 0.05). Rumen microbial diversity was significantly higher in the FAM group (*p* < 0.05). At the genus level, the FAM group showed enrichment of fiber-degrading bacteria, including *Rikenellaceae_RC9_gut_group*, *unclassified_Muribaculaceae*, and *unclassified_F082*. In newborn lambs, plasma GH and IGF-1 levels increased markedly in both the FAM and AM groups (*p* < 0.01). In the FAM group, immunoglobulin A (IgA) and immunoglobulin G (IgG) concentrations were also markedly elevated (*p* < 0.01). In addition, lambs in the FAM group exhibited substantially higher plasma catalase (CAT) activity and nitric oxide (NO) concentration (*p* < 0.01), whereas plasma total antioxidant capacity was significantly lower (*p* < 0.01). In conclusion, AM and FAM supplementation increased the relative abundance of Bacteroidetes, regulated fatty acid synthesis through modulation of plasma metabolites, enhanced antioxidant capacity in ewes, increased milk fat percentage, and improved immune function, antioxidant status, and growth performance in lambs. To provide a theoretical basis and data support for its application in ewes after lambing and for improving the growth and development of lambs. Overall, FAM produced superior effect.

## 1. Introduction

The sheep industry occupies a critical role in China’s livestock economy. Turpan black sheep, an indigenous breed unique to Xinjiang, exhibit strong roughage tolerance, excellent adaptability, and high meat quality, making them an important source of income for local herders [1]. Previous studies have shown that postpartum females are highly susceptible to negative energy balance and immunosuppression, frequently accompanied by reduced feed intake, aggravated oxidative stress, and elevated risk of mammary infection. These challenges can result in insufficient milk yield and compromised milk quality, thereby impairing lamb growth and development [2]. Effective postpartum nutritional management is therefore essential for restoring ewe health and improving lactation performance.

As a traditional tonic herb in Chinese medicine, Astragalus has had more than 100 active components isolated and identified, primarily consisting of Astragalus polysaccharides, saponins, and flavonoids. It exhibits diverse biological activities, including immunomodulation, antioxidant capacity, anti-inflammatory effects, and regulation of nutrient metabolism [3,4]. Consistent with the anti-inflammatory and tissue-protective properties of natural plant polysaccharides, Poria cocos polysaccharide could effectively alleviate inflammatory injury induced by pathogenic bacteria via regulating inflammatory response pathways [5]. Research has confirmed that Astragalus possesses high feed value in animal husbandry; in addition to regulating growth performance, it can also modulate the microbial balance in the rumen of ruminants and enhance the animals’ resistance [6]. However, traditional Astragalus suffers from drawbacks such as low bioavailability of its active components and slow onset of action [7]. Fermentation with beneficial microorganisms can enhance the bioactivity of AM polysaccharides, astragalosides, and flavonoids, thereby maximizing biological effects [8]. The majority of existing investigations on herbal feed additives have centered on monogastric livestock, notably pigs, poultry, and dairy cattle. As ruminants, sheep typically demonstrate greater efficiency in utilizing botanical supplements than monogastric species. This study utilized postpartum Turpan black ewes (*Ovis aries*) as experimental subjects to conduct a comprehensive comparison of dietary AM versus FAM supplementation. Maternal outcomes assessed included lactation performance, rumen microbial community architecture, antioxidant defense, and plasma metabolic fingerprints. Offspring outcomes encompassed growth trajectories, immunological function, and oxidative balance. The research sought to unravel the mechanistic pathways through which FAM regulates maternal physiological status and milk secretion, and to elucidate how maternal FAM intake translates into developmental benefits for lambs via lactational transfer. Ultimately, this work provides a scientifically grounded rationale for the wider and more judicious deployment of FAM in sheep husbandry.

## 2. Materials and Methods

### 2.1. Institutional Review Board Statement

The research protocol was authorized by the Institutional Animal Ethics Committee of Xinjiang Agricultural University (Reference: 2020032, 7 May 2020; 2020024, 20 March 2020). Animal handling, feeding, and sampling were executed following the institutional protocols and prevailing legal frameworks governing animal research.

### 2.2. Experimental Materials

AM and FAM powders—identical to those characterized in our prior study—were supplemented at 2 g/ewe/d. Raw AM (yellow powder, Weishuihong Cooperative, Dingxi, China) served as the substrate for FAM (Heilongjiang Lingkang Biotechnology Co., Ltd., Qiqihar, China), which contained ≥1 × 10^9^ CFU/g of both B. subtilis and B. licheniformis. Dosage selection followed manufacturer guidelines and interspecies conversion protocols [9]. Prior research by Han et al. has demonstrated that dietary inclusion of 2% unfermented or fermented AM effectively enhances growth performance and ameliorates oxidative stress in broilers, thereby validating the biological efficacy of this dosage range in monogastric species [10].

All double-antibody sandwich ELISA detection kits adopted in this study matched the identical batch of reagents used in our prior experiment, and these products were purchased from Shanghai Yuanju Biotechnology Center [9]. The corresponding product serial numbers are listed below: Insulin–like Growth Factor–1 (IGF-1), YJ10602–1; Growth Hormone (GH), Y7710681–1; Immunoglobulin A (IgA), YJ440029–1; Immunoglobulin M (IgM), YJ440024; and Immunoglobulin G (IgG), YJ440021–1. Antioxidant assay kits were obtained from Shanghai Enzyme–linked Biotechnology Co., Ltd., Shanghai, China, with kit numbers as follows: Total Antioxidant Capacity (T-AOC), W96–M (1720); Catalase (CAT), W96–N (1620); Superoxide Dismutase (SOD), W96–N (1733); Glutathione Peroxidase (GSH–Px), W96–N (1047); Malondialdehyde (MDA), W96–N (1620); and Nitric Oxide (NO), W96–N (1620).

### 2.3. Experimental Site

The trial was conducted at Huishang Ecological Animal Husbandry Co., Ltd., Toksun County, Turpan City, Xinjiang, China, from June to July 2025 (87°14′05″–89°11′08″ E, 41°21′14″–43°18′11″ N). The experimental period lasted 45 days.

### 2.4. Experimental Design and Grouping

**Figure 1:**
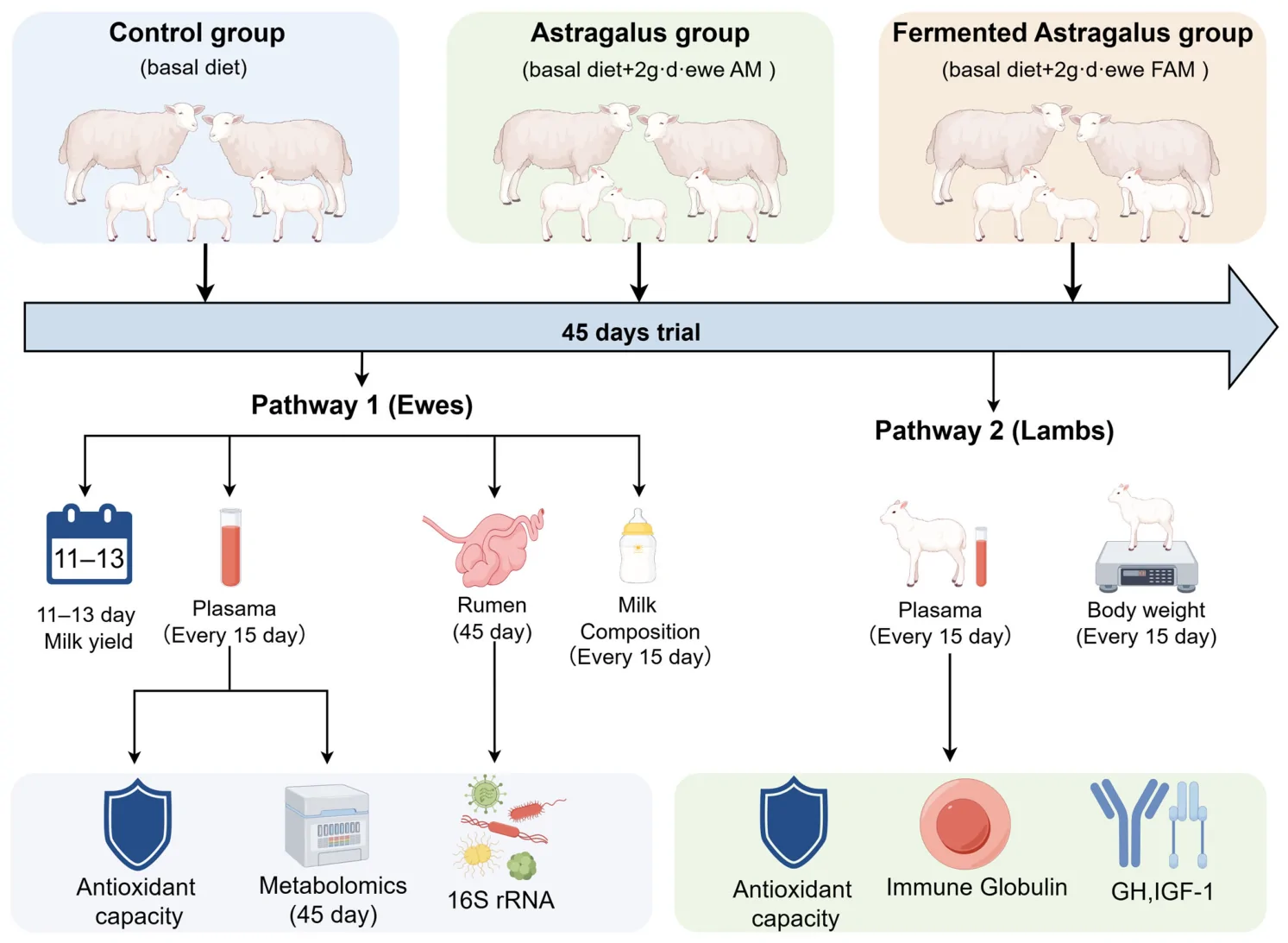
Experimental Design Diagram.

Forty–five postpartum ewes with comparable body weight, identical parity, similar post-synchronization parturition timing, single-lamb delivery, and similar lamb birth weight were selected and randomly assigned to three groups (*n* = 15/group): Control group (basal diet), AM group (+2 g AM/ewe/day), and FAM group (+2 g FAM/ewe/day). All ewes were maintained under identical feeding conditions from the day of parturition to the end of the trial. The Control group received the basal diet alone; the AM group received the basal diet plus 2 g/ewe/day AM; and the FAM group received the basal diet plus 2 g/ewe/day FAM through day 45 postpartum (Figure 1).

### 2.5. Animals and Management

All ewes were group-housed in a single barn throughout the trial, with 15 animals per pen. The 45-day experimental period commenced at parturition and extended to 45 days postpartum. During this interval, ewes received a total mixed ration (TMR) delivered by automated feeding vehicle at 10:30 and 19:30 daily. The TMR was supplied by Huishang Ecological Animal Husbandry Co., Ltd. (Turpan City, Xinjiang, China). Feed and water were available ad libitum. Ewes in the Control group were supplemented with 500 g wheat bran per head daily. For the AM and FAM groups, 2 g/ewe/day of the respective additive was premixed with wheat bran and placed in the feeding trough; only upon complete consumption of this supplemented bran was the routine TMR ration offered. Dietary composition and nutrient specifications are detailed in Table 1. Lambs were maintained with their dams in the same facility. Concentrate pellet introduction began at 15 days of age, with both feed and water provided ad libitum. The basal diet nutrient composition was formulated in strict accordance with the Feeding Standard of Meat-producing Sheep and Goats (NY/T 816-2021) issued by the Ministry of Agriculture and Rural Affairs of the People’s Republic of China, combined with the actual physiological status in this trial [11].

The identical lamb basal starter feed formula reported in our previous study was adopted in this trial [9]. No extra functional additives were added to the basal starter feed during the whole feeding period.

### 2.6. Sample Collection

#### 2.6.1. Feed Sample

Feed sampling was conducted on experimental days 22 through 26, with collections performed both prior to and following the morning and evening feeding sessions. Pre-feeding samples were obtained by extracting 100 g of fresh ration from 5–8 randomly selected locations within the trough. Post-feeding samples comprised the residual feed remaining in the trough. All collected materials were homogenized on clean trays and consecutively quartered to yield representative subsamples of 300–500 g. These subsamples were immediately sealed, labeled with the collection date, and reserved for subsequent moisture and nutrient composition analyses.

#### 2.6.2. Milk Sample

Milk yield was measured for all 15 ewes per group by hand-milking at 09:00 and 19:00 on postpartum days 11, 12, and 13. For milk composition analysis, six ewes per group were randomly selected and sampled on days 0, 15, 30, and 45 postpartum. On composition sampling days, the selected ewes were milked twice daily (09:00 and 19:00) following a standardized procedure: teat cleaning and disinfection, discarding of foremilk, and collection of 30–50 mL fresh milk into 30 mL bottles. Samples from both milking sessions were homogenized, pooled, and stored at −20 °C until analysis.

#### 2.6.3. Blood Sample Collection

Six Turpan black ewes were randomly selected from each group. Jugular venous blood was collected into sodium heparin anticoagulant tubes before morning feeding on postpartum days 0, 15, 30, and 45. At the same time points after birth, jugular blood was also collected from lambs born to the selected ewes. All blood samples were centrifuged at 3500 rpm for 10 min using a refrigerated high-speed centrifuge (Model 5427R, Eppendorf, Hamburg, Germany) to separate plasma. Plasma aliquots were transferred into 1.8 mL cryotubes, snap-frozen in liquid nitrogen, and stored at −80 °C for subsequent untargeted metabolomics, hormone, and antioxidant–capacity analyses.

#### 2.6.4. Rumen Fluid

On postpartum day 45, rumen fluid was collected from six fixed ewes per group using an ovine stomach–tube sampler. Approximately 100 mL rumen fluid per ewe was filtered through four layers of sterile gauze and aliquoted into 5 mL cryotubes. All samples were immediately frozen in liquid nitrogen and stored at −80 °C for subsequent 16S rRNA gene sequencing.

### 2.7. Sample Determination

#### 2.7.1. Determination of Feed Samples

The dry matter (DM) content of the diet was determined by oven drying at 105 °C in accordance with Chinese national standard GB/T 6435-2014 [12]. Crude ash content was measured following GB/T 6438-2025 [13]. Crude protein was quantified via the Kjeldahl method (AOAC 990.03) [14]. Calcium was determined using the o-cresolphthalein complexone colorimetric method (AOAC 968.08), and phosphorus was measured via ammonium vanadate molybdate colorimetry (AOAC 965.17) [15,16]. Neutral detergent fiber (NDF, AOAC 2002.04) and acid detergent fiber (ADF, AOAC 973.18) were sequentially determined [17,18].

#### 2.7.2. Determination of Ewe Milk Yield

Ewe milk yield was estimated from lamb body-weight differences before and after suckling [19]. During the measurement period, lambs received only maternal milk. Milk yield was measured in fifteen selected ewes per group on postpartum days 11–13. Lambs were separated from ewes at 7:30, weighed before each suckling session at 3 h intervals, allowed to suckle for 10–15 min, then immediately reweighed after separation. The pre–to post–suckling weight gain was recorded as milk intake per session. Suckling sessions were scheduled at 10:30, 14:00, 17:30, and 21:00. The sum of four session values was defined as daily milk yield per ewe.

#### 2.7.3. Determination of Milk Components

After thawing at room temperature, milk samples were preheated in a 40 °C water bath. Conventional milk components, including milk fat, milk protein, lactose, non–fat solids, and ash, were determined with an automatic milk composition analyzer (MilkoScan FT3, FOSS, Hilloeroed, Denmark) according to standard operating procedures.

#### 2.7.4. Determination of Lamb Growth Performance

Lamb birth weight was recorded. Body weight was measured every 15 days before morning feeding. The high-precision electronic scale used to record lamb birth weight and body weight at each sampling time point is Lufeng, manufactured in Huizhou, Guangdong Province, China. And total weight gain and average daily gain (ADG) were calculated as follows:

Total weight gain=Final body weight−Initial body weight

ADG=(Final body weight−Initial body weight)/days

#### 2.7.5. Determination of Plasma Samples

GH, IGF-1, IgA, IgG, IgM, T-AOC, CAT, SOD, GSH–Px, MDA, and NO were quantified using the corresponding commercial assay kits. Plasma collected from ewes on postpartum day 45 was submitted to Novogene Co., Ltd. (Tianjin, China) for untargeted metabolomics analysis.

#### 2.7.6. Analysis of Rumen Fluid Samples

On postpartum day 45, rumen fluid was collected from experimental ewes and submitted to Novogene Co., Ltd. (Beijing, China) for 16S rRNA gene sequencing. The V3–V4 hypervariable region of the bacterial 16S rRNA gene was amplified by PCR using universal primers 314F (5′-CCTAYGGGRBGCASCAG-3′) and 806R (5′-GGACTACNNGGGTATCTAAT-3′). Amplicons were verified by 2% agarose gel electrophoresis, purified with magnetic beads, and recovered for library construction. Paired-end sequencing was performed on the Illumina NovaSeq platform (San Diego, America). Raw sequences were processed using QIIME2 and visualized on the NovoMagic platform (Tianjin, China).

### 2.8. Statistics

Microsoft Excel was used for initial data compilation. Milk yield was analyzed by one-way ANOVA followed by Duncan’s test in SPSS 27.0; a mixed model for repeated measures in SAS 9.4 was applied to all other variables (milk composition, antioxidants, lamb growth, hormones, immunity). Values denote mean ± SEM.

Novogene’s cloud platform processed metabolomic and microbiome data. Correlations among differential metabolites, rumen bacteria, and milk traits were computed in R 4.5.2. GraphPad Prism 9.5 and Adobe Illustrator 2025 generated the figures. *p* < 0.05 and *p* < 0.01 were taken as significant and highly significant, respectively.

## 3. Results

### 3.1. Dietary AM and FAM in Postpartum Ewes: Multisystemic Outcomes

#### 3.1.1. Milk Yield Response to Astragalus and Fermented Astragalus in Postpartum Ewes

**Figure 2:**
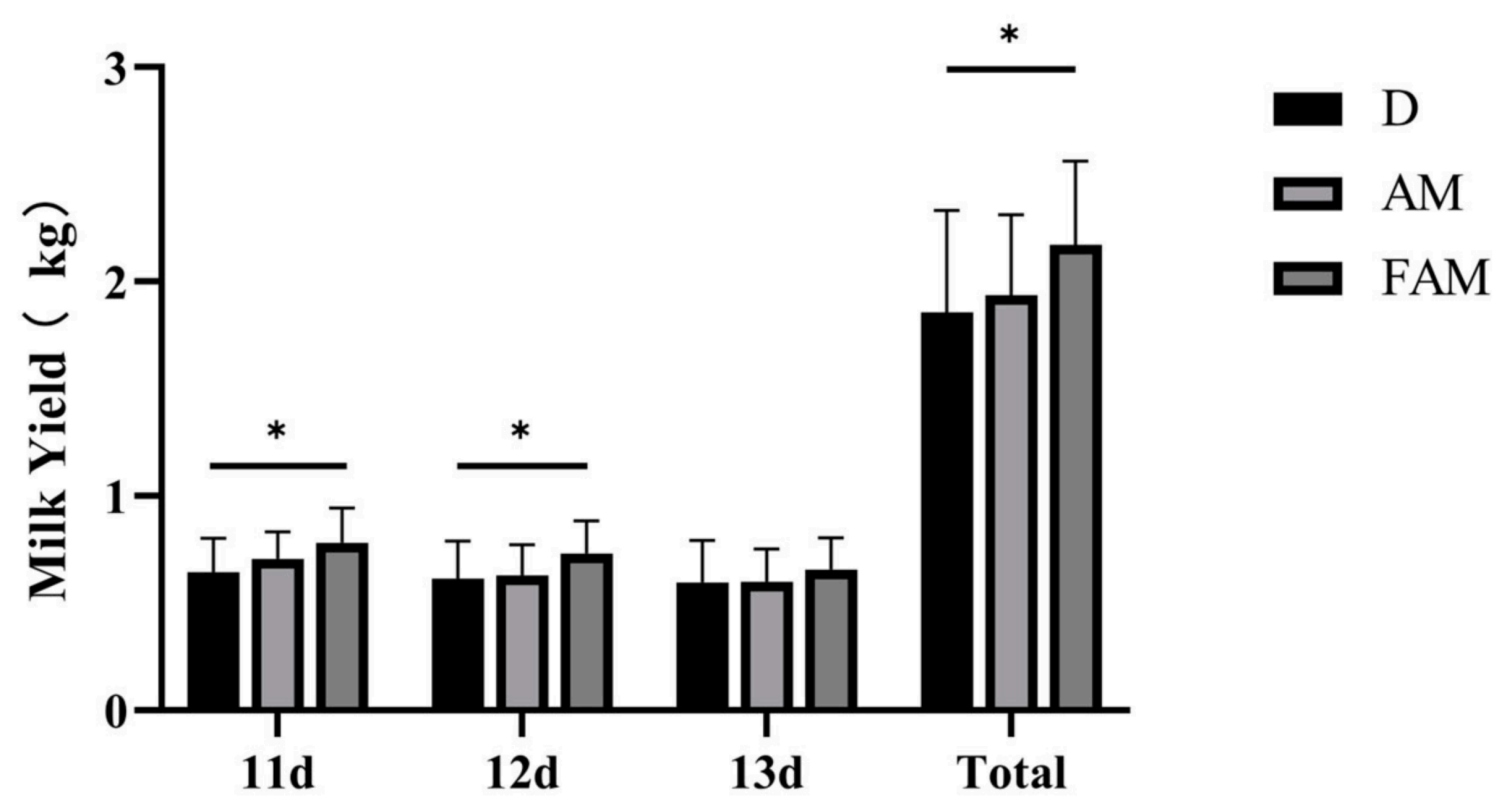
Effects of AM and FAM on Milk Yield in Postpartum Ewes (*n* = 15, kg). Note: An asterisk (*) signals distinct statistical outcomes (*p* < 0.05); unmarked comparisons show indistinguishable group results (*p* > 0.05). Graphic marker definitions: D = control cohort, AM = raw astragalus group, FAM = fermented astragalus group. This labeling convention is uniformly used for all later figures.

As shown in Figure 2, compared with the Control group, milk yield in the AM group increased by 10.94% on postpartum day 11 (*p* > 0.05), whereas milk yield in the FAM group increased significantly by 21.88% (*p* < 0.05). On postpartum day 12, milk yield in the FAM group remained significantly higher, with a 19.67% increase (*p* < 0.05). On postpartum day 13, milk yield in the FAM group increased by 10%, but no significant difference was observed among groups (*p* > 0.05). Overall, total milk yield from postpartum days 11 to 13 was significantly greater in the FAM group, with a 16.67% increase (*p* < 0.05). Supplementation with FAM significantly elevated daily milk production on days 11 and 12 after delivery and improved cumulative milk yield over the three-day period, while AM treatment failed to produce obvious promoting effects on lactation performance.

#### 3.1.2. Milk Composition in Response to AM and FAM Supplementation

Variations of milk fat ratio related to treatment, sampling time and their interaction are summarized in Table 2. Single factors of different herbal additives and detection time both induced highly prominent changes in this milk composition indicator (*p* < 0.01). Compared with sheep receiving unfermented astragalus and blank basal diet, animals supplied with fermented astragalus obtained far superior milk fat content (*p* < 0.01), and the cross-effect between additive type and sampling time reached a non-significant level (*p* > 0.05). For milk protein percentage, only the time effect was extremely significant (*p* < 0.01), with no significant group or interaction effects (*p* > 0.05). For lactose percentage, ash content, and solid-not-fat (SNF), group and interaction effects were not significant (*p* > 0.05), whereas time effects were extremely significant (*p* < 0.01). Treatment only altered milk fat content, with FAM notably raising milk fat percentage. The dynamic changes of milk protein, lactose, ash and SNF were merely driven by lactation stage, and no cross interaction existed between treatment and sampling time for all milk composition indicators.

#### 3.1.3. Lactating Sheep Plasma Antioxidant Responses to Raw and Fermented Herbal Astragalus Additives

As shown in Table 3, FAM supplementation extremely significantly increased ewe plasma T-AOC, with significant group, time, and group × time interaction effects (*p* < 0.01). Plasma CAT and SOD showed extremely significant time effects only (*p* < 0.01), with no significant group or interaction effects (*p* > 0.05). Plasma GSH-Px showed a significant group effect (*p* < 0.05), with values in the FAM group significantly higher than in the Con group (*p* < 0.05), along with an extremely significant time effect (*p* < 0.01) and a non-significant interaction effect (*p* > 0.05). For plasma NO and MDA, both group and time effects were extremely significant (*p* < 0.01). Compared with the Con group, both indices were extremely significantly lower in the FAM group (*p* < 0.01), while the interaction effect was not significant (*p* > 0.05). FAM treatment improved overall plasma antioxidant capacity by elevating T-AOC and GSH-Px while reducing oxidative products NO and MDA. CAT and SOD levels only changed over time and were unaffected by supplementation, and no interaction between group and time was observed for most antioxidant indicators except T-AOC.

#### 3.1.4. Effects of Astragalus and Fermented Astragalus Supplementation on Plasma Metabolites of Postpartum Ewes

##### Venn Analysis of Differential Metabolites

**Figure 3:**
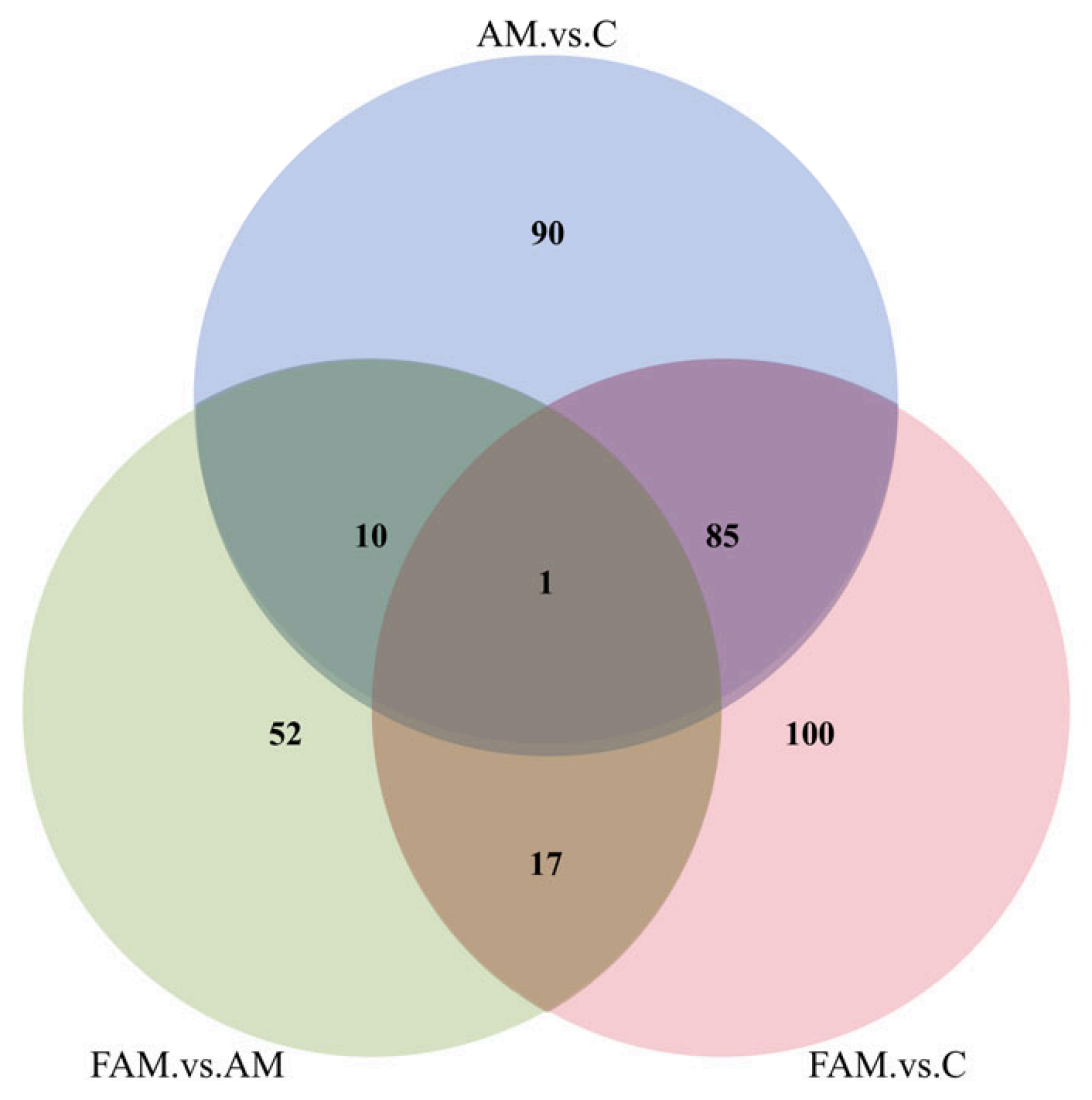
Venn Analysis of plasma differential metabolites. Note: AM vs. C represents the comparison between AM group and Con group, FAM vs. C stands for FAM group and Con group, and FAM vs. AM indicates FAM group and AM group.

As shown in Figure 3, plasma metabolites were classified based on sequencing data and compositional similarity. Between the Control vs. AM and Control vs. FAM comparisons, 217 distinct metabolites were identified. Specifically, 186 differential metabolites were detected in Control vs. AM, 203 in Control vs. FAM, and 86 were shared between these two comparisons. Between Control vs. AM and AM vs. FAM, 244 distinct metabolites were identified, including 186 in Control vs. AM, 80 in AM vs. FAM, and 11 common metabolites. Between AM vs. FAM and Control vs. FAM, 247 distinct metabolites were identified, including 80 in AM vs. FAM, 203 in Control vs. FAM, and 18 overlapping metabolites.

##### Partial Least Squares-Discriminant Analysis (PLS–DA) Among Groups

**Figure 4:**
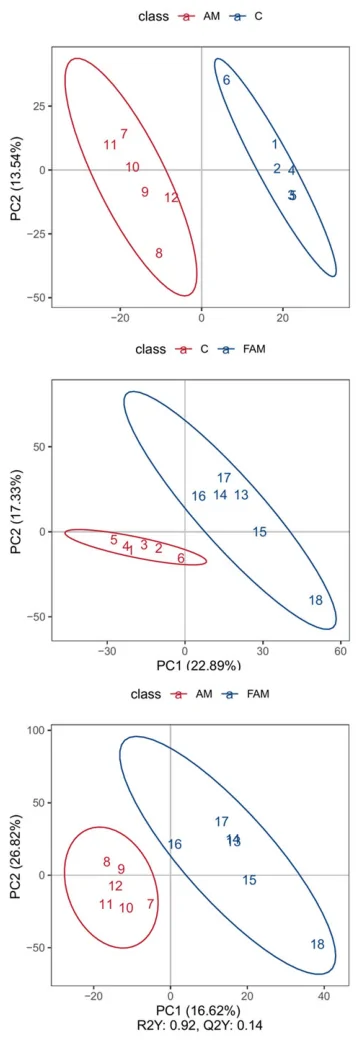
PLS-DA Analysis of plasma differential metabolites.

The results are presented in Figure 4, the PLS–DA score plot was used to visualize model classification performance. Greater separation among sample clusters indicates stronger discrimination. Clear separation was observed among all groups, indicating good classification performance.

##### Differential Metabolite Profiling: Conotrol Group Versus AM Group

**Figure 5:**
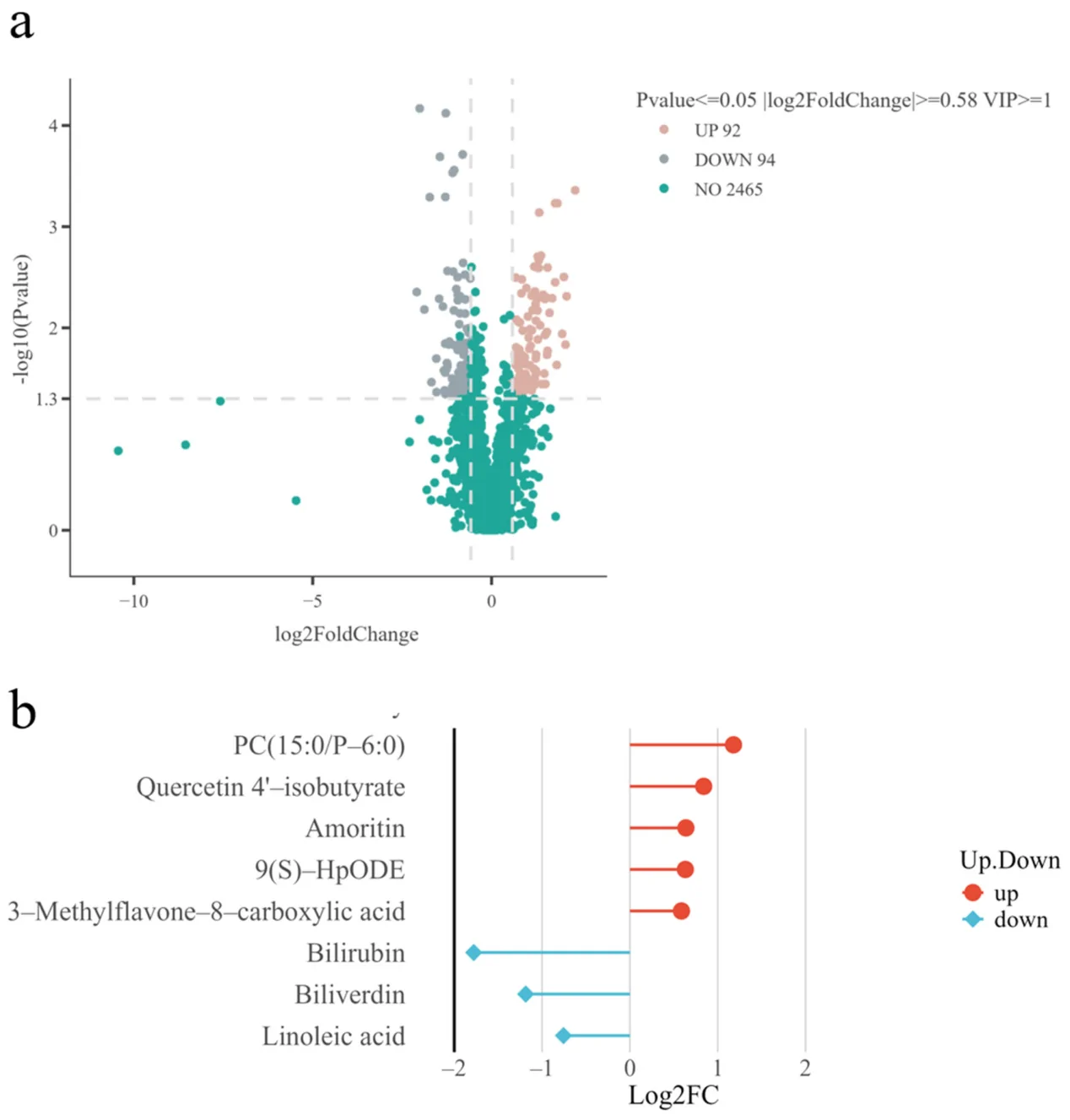
Volcano plots (**a**) and matchstick plots (**b**) of differentially expressed metabolites in the Control and AM groups. Note: Every plotted dot corresponds to an independent metabolic compound within this dataset. Spots marked in red denote substances with remarkably elevated expression levels, while blue-labeled points signify metabolic compounds showing obvious decline in abundance. Gray graphic markers refer to metabolites lacking statistically notable expression shifts. The size of dots reflects the VIP value; a higher VIP value means a greater contribution of the metabolite to group classification. Stem-leaf plots can visually present the up–regulation or down–regulation trends of differential metabolites, especially those with large fold changes. In the plot, red stems represent upregulated metabolites and blue stems represent downregulated ones. The length of the stem corresponds to the value of log_2_(Fold Change), namely longer stems indicate larger fold changes. In addition, the dot size represents the VIP value.

As shown in Figure 5a, 2651 plasma metabolites were detected in Control vs. AM. Among these, 186 metabolites (7.02%) were significantly changed. Of the differential metabolites, 92 were significantly upregulated (*p* < 0.05), accounting for 3.55% of total metabolites and 50.54% of differential metabolites, while 94 were significantly downregulated (*p* < 0.05), accounting for 3.47% of total metabolites and 49.46% of differential metabolites. The remaining 2465 metabolites showed no significant difference (*p* > 0.05). Further screening showed that 9(S)–HpODE, quercetin 4′–isobutyrate, amoritin, and 3–methylflavone–8–carboxylic acid were significantly upregulated (*p* < 0.05), whereas biliverdin, bilirubin, and linoleic acid were significantly downregulated (*p* < 0.05) (Figure 5b).

##### Comparative Metabolite Profiling: The Control Group and the FAM Group

**Figure 6:**
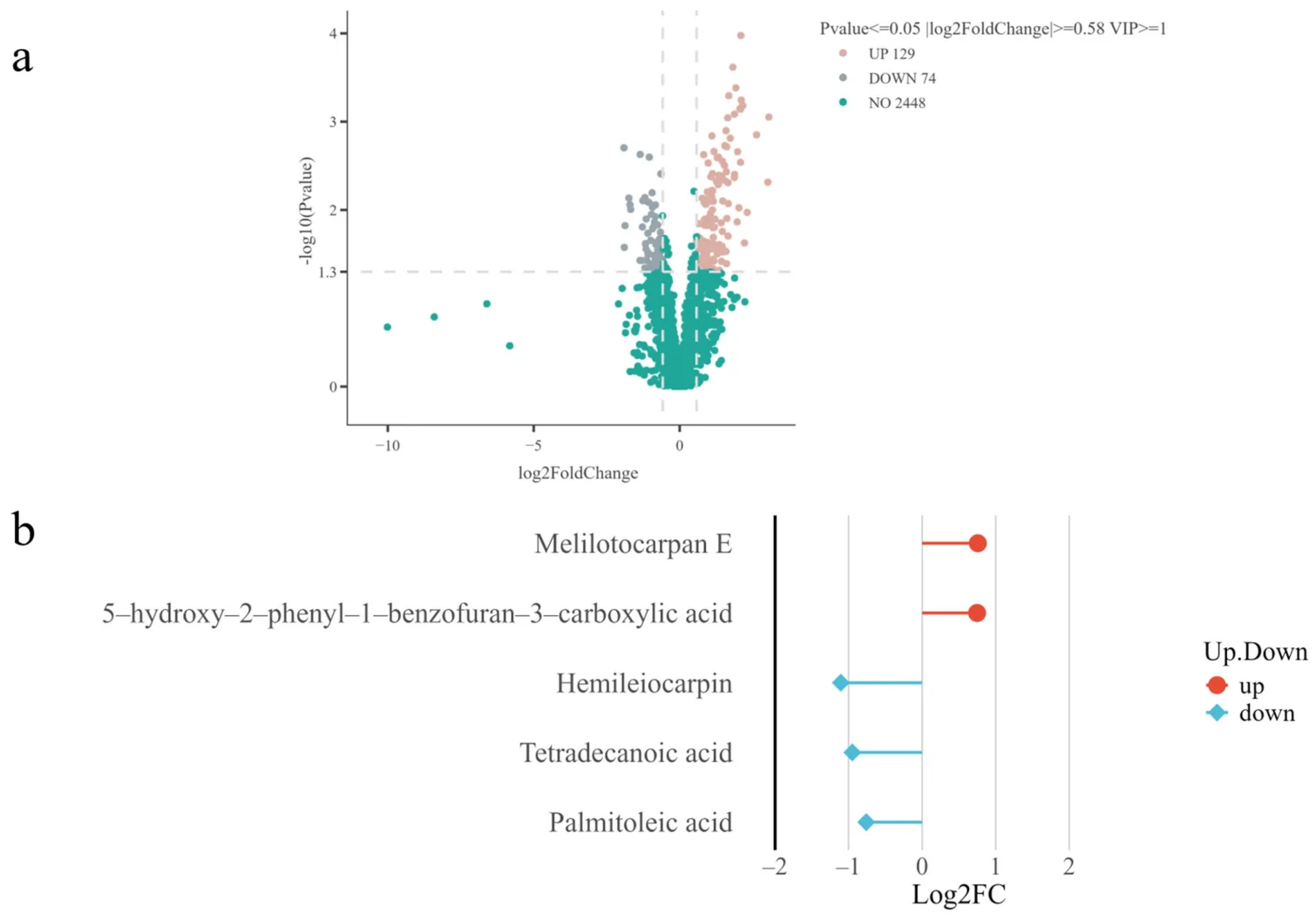
Volcano plots (**a**) and matchstick plots (**b**) of differentially expressed metabolites in the Control and FAM groups. Note: Group definitions are the same as Figure 5.

As shown in Figure 6a, a total of 2651 plasma metabolites were identified in the Control vs. FAM comparison. Among these, 203 metabolites were significantly altered, accounting for 7.66% of all detected metabolites. Specifically, 129 metabolites were significantly upregulated (*p* < 0.05), representing 4.86% of total metabolites and 63.55% of all differential metabolites, while 74 metabolites were significantly downregulated (*p* < 0.05), representing 2.79% of total metabolites and 36.45% of all differential metabolites. The remaining 2448 metabolites showed no significant differences (*p* > 0.05). Further screening showed that melilotocarpan E and 5-hydroxy-2-phenyl-1-benzofuran-3-carboxylic acid were significantly upregulated (*p* < 0.05), whereas hemileiocarpin, tetradecanoic acid, and palmitoleic acid were significantly downregulated in plasma (*p* < 0.05) (Figure 6b).

##### Comparative Metabolite Profiling: The AM Group and FAM Group

**Figure 7:**
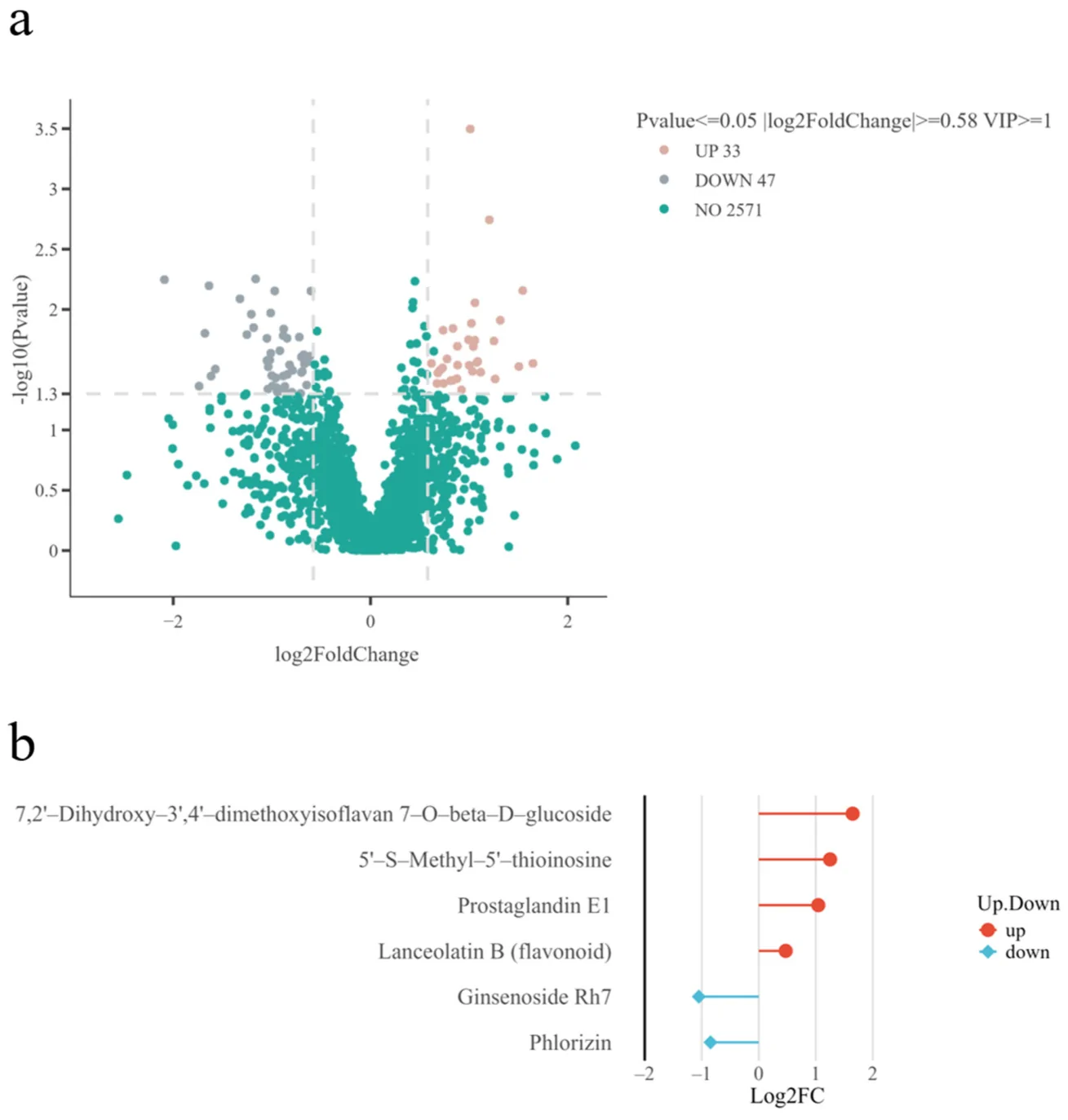
Volcano plots (**a**) and matchstick plots (**b**) of differentially expressed metabolites in the AM and FAM groups. Note: Group definitions are the same as Figure 5.

As shown in Figure 7a, a total of 2651 plasma metabolites were detected in the AM vs. FAM comparison. Among these, 80 metabolites were significantly altered, accounting for 3.02% of all detected metabolites. Specifically, 33 metabolites were significantly upregulated (*p* < 0.05), representing 1.24% of total metabolites and 41.25% of all differential metabolites, while 47 metabolites were significantly downregulated (*p* < 0.05), representing 1.77% of total metabolites and 58.75% of all differential metabolites. The remaining 2571 metabolites showed no significant differences (*p* > 0.05). Further screening indicated that 7,2′–dihydroxy–3′,4′-dimethoxyisoflavan 7–O–beta–D–glucoside, 5′–S-methyl–5′–thioinosine, prostaglandin E1, and lanceolatin B (flavonoid) were significantly upregulated (*p* < 0.05), whereas ginsenoside Rh7 and phlorizin were significantly downregulated in plasma (*p* < 0.05) (Figure 7b).

##### KEGG Pathway Enrichment Analysis of Differential Metabolites

**Figure 8:**
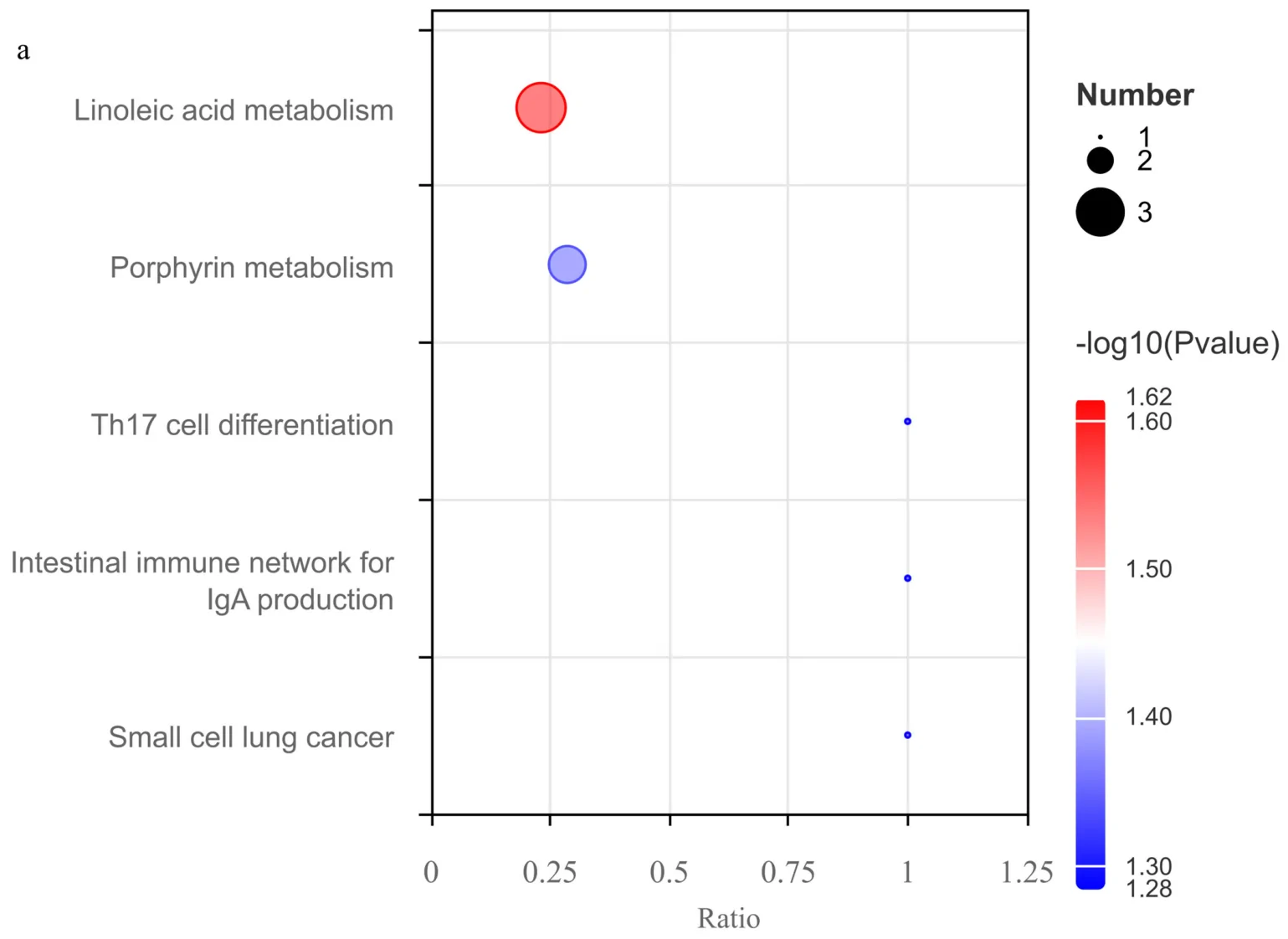

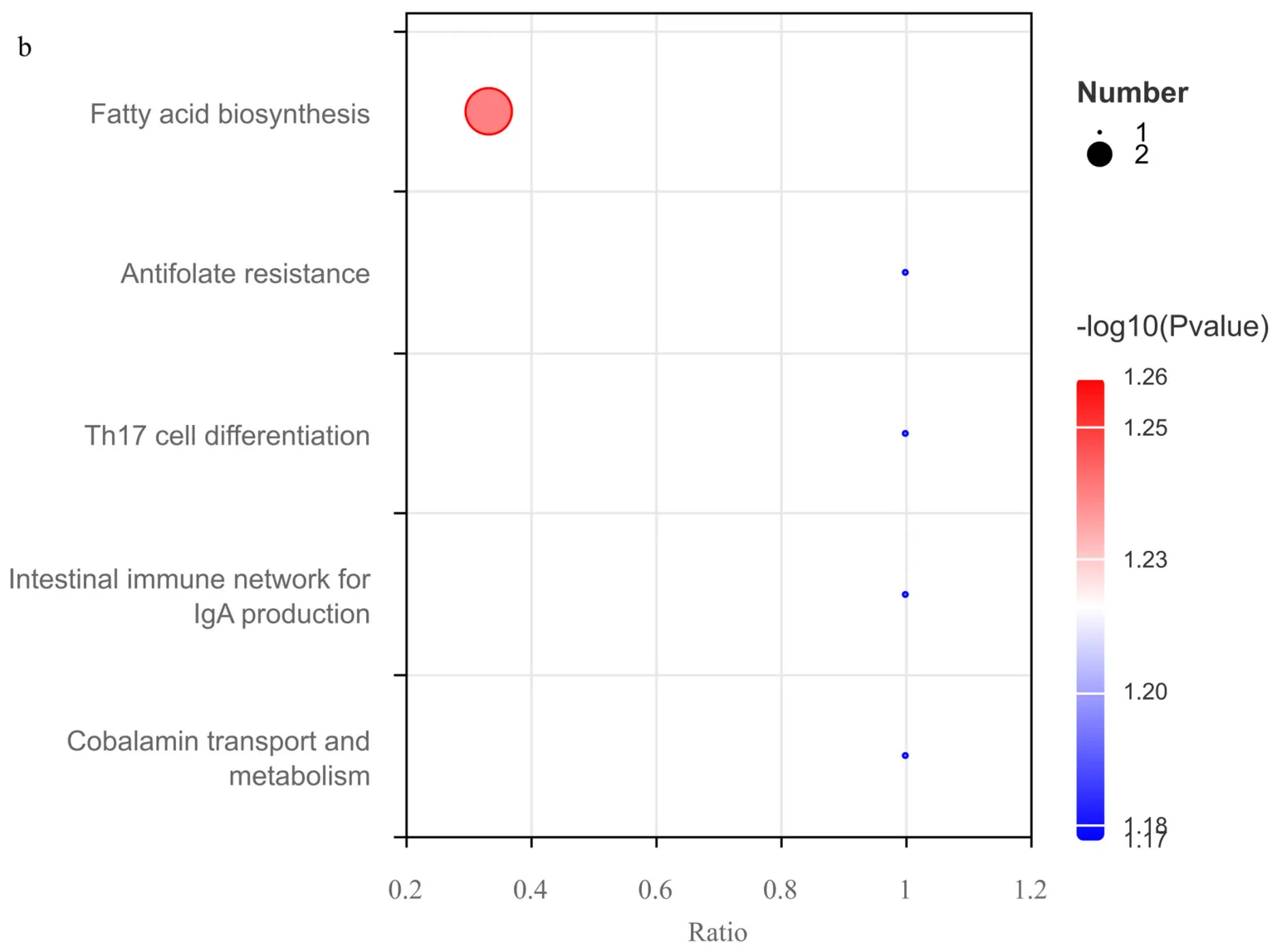

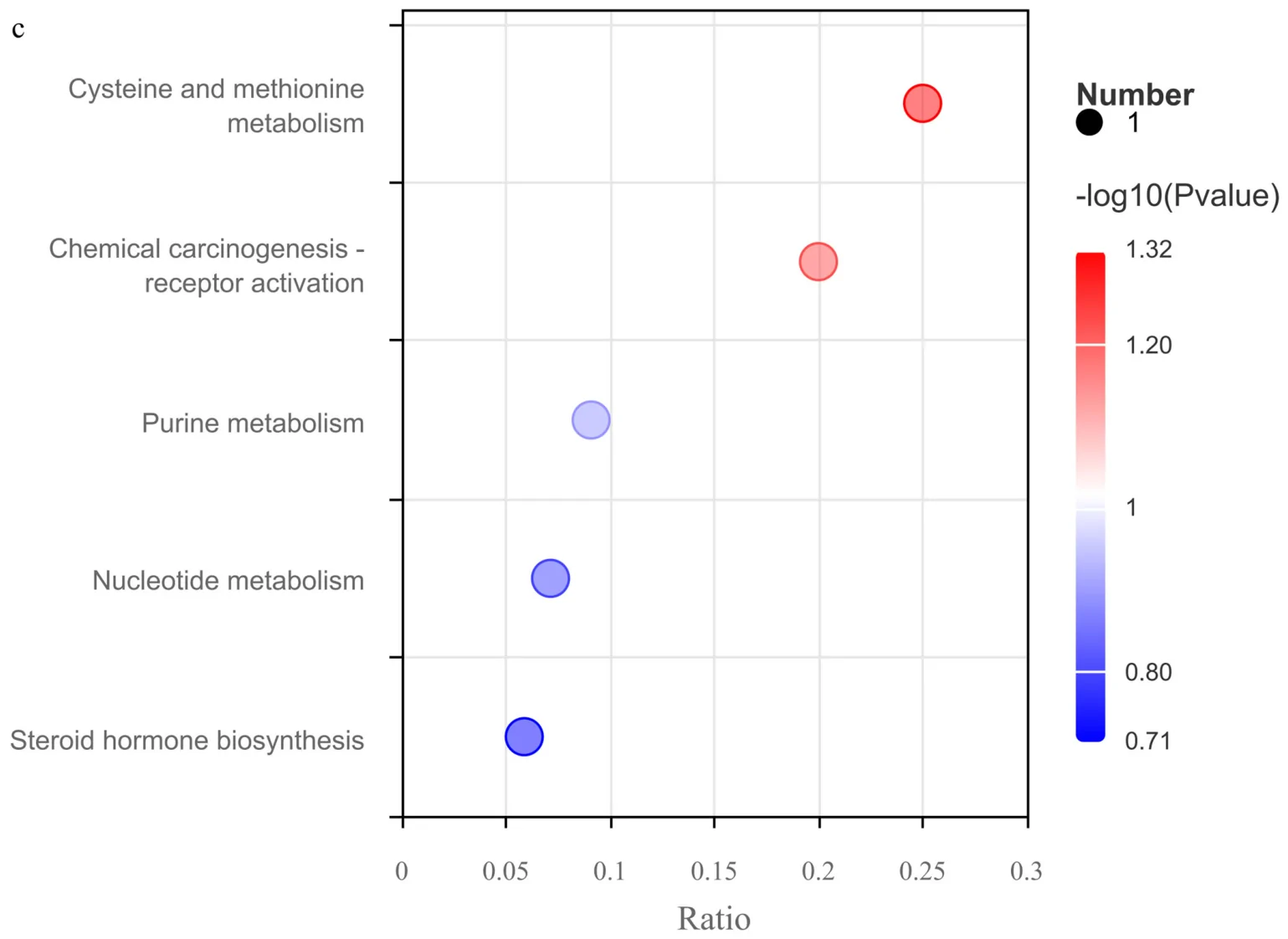
(**a**) KEGG pathway enrichment bubble plot comparing the Control group and the AM group; (**b**) KEGG pathway enrichment bubble plot comparing the Control group and the FAM group; (**c**) KEGG pathway enrichment bubble plot comparing the AM group and the FAM group. Note: The top 5 KEGG enriched pathways ranked by ascending *p*–value are displayed in the figure. The x–axis represents the ratio of differential metabolites to total metabolites within each pathway; higher values indicate stronger enrichment. Dot color corresponds to the hypergeometric test *p*-value (redder color indicates smaller *p*-value, higher reliability, and stronger statistical significance). Dot size indicates the number of differential metabolites in each pathway, with larger dots representing more differential metabolites.

KEGG–based screening showed that the key differential pathways for Control vs. AM were linoleic acid metabolism and porphyrin metabolism (Figure 8a). For Control vs. FAM, fatty acid biosynthesis was the predominant differential pathway (Figure 8b). For AM vs. FAM, cysteine and methionine metabolism was the major enriched pathway (Figure 8c).

#### 3.1.5. Rumen Microflora Alterations in Lactating Sheep Receiving Raw and Fermented Herbal Astragalus Preparations

##### Venn Analysis of Rumen Microbial Community Composition

**Figure 9:**
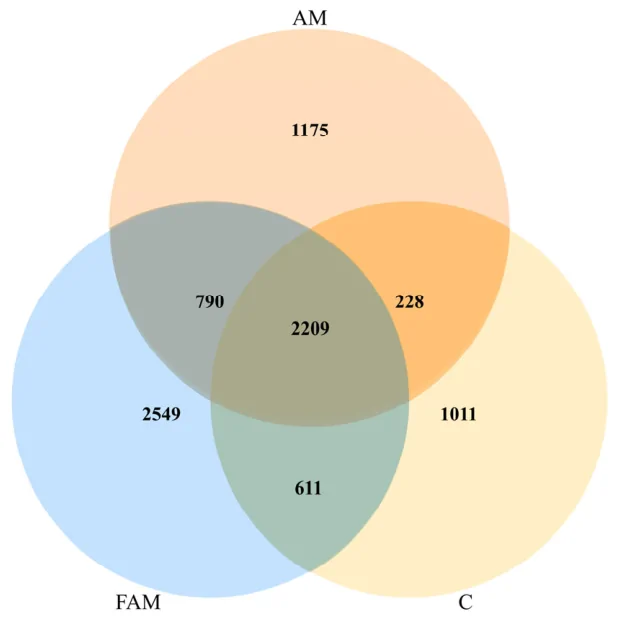
Rumen Microbial Diversity Influences the Venn Diagram.

As shown in Figure 9, a Venn diagram based on OTUs was used to characterize rumen microbial communities in sheep supplemented with FAM or FAM. The numbers of shared and unique OTUs were clearly distinguished among groups. A total of 2209 OTUs were shared by all three groups. Group–specific OTUs were 1011 in Control, 1175 in AM, and 2549 in FAM.

##### Analysis of Alpha Diversity

As shown in Table 4, no significant differences were detected among Control, AM, and FAM in Features, Pielou_e, Dominance, or Simpson indices (*p* > 0.05). In contrast, the FAM group showed extremely significant differences in Chao1, Good’s coverage, and Shannon indices (*p* < 0.01), indicating that FAM markedly affected rumen microbial diversity in ewes. Species coverage for all samples reached 99.9%, suggesting that sequencing depth was sufficient to capture the true rumen microbial composition.

##### Principal Coordinates Analysis (PCoA)

**Figure 10:**
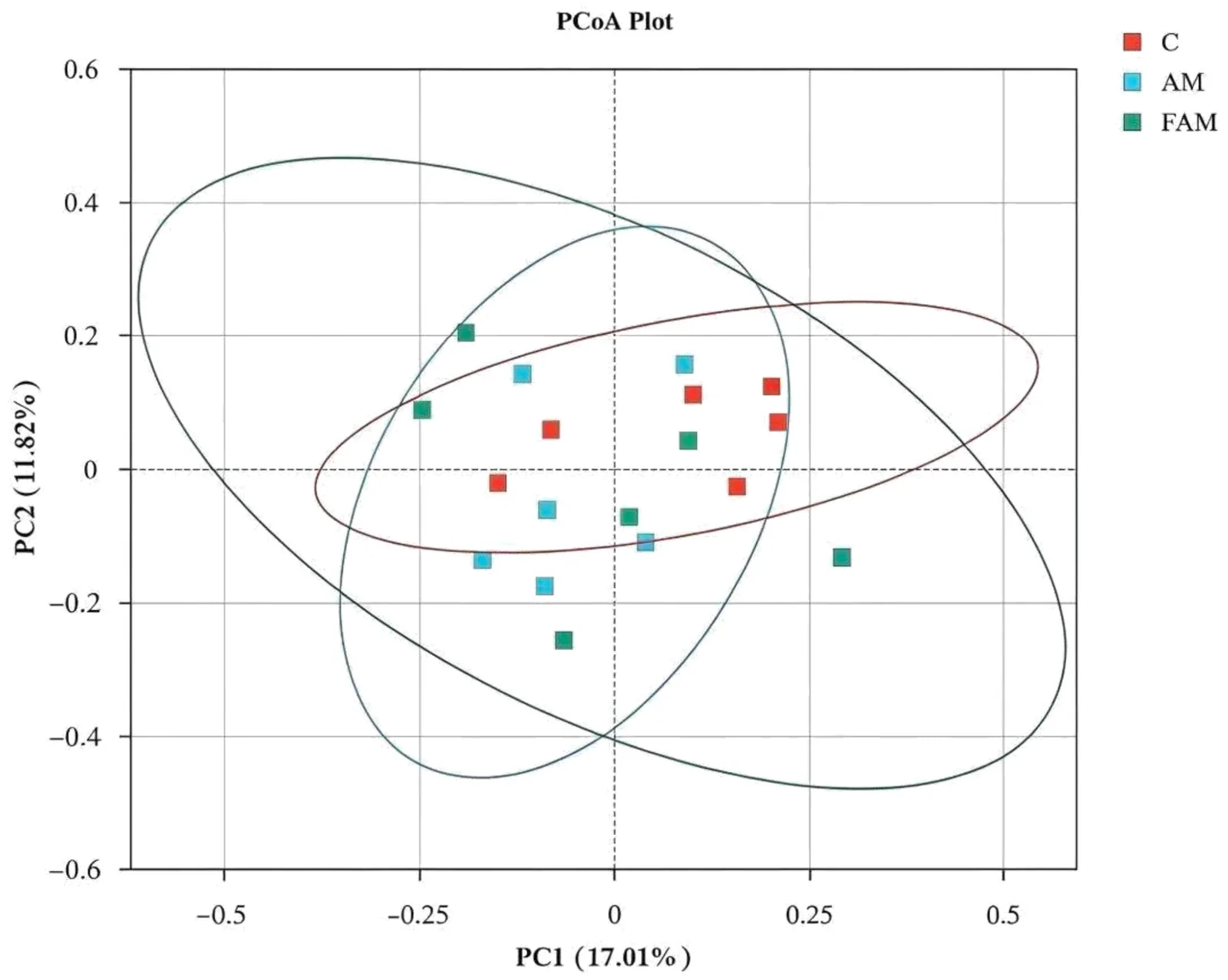
PCoA analysis of rumen microbiota in ewes supplemented with Astragalus and fermented Astragalus.

As shown in Figure 10, PCoA was used to visualize microbial community grouping. Greater distances between sample clusters indicate stronger between-group differentiation. Clear separation among treatment groups was observed, indicating a robust grouping pattern. The first principal component explained 17.01% of total variation, and the second explained 11.82%.

##### Beta Diversity Analysis

**Figure 11:**
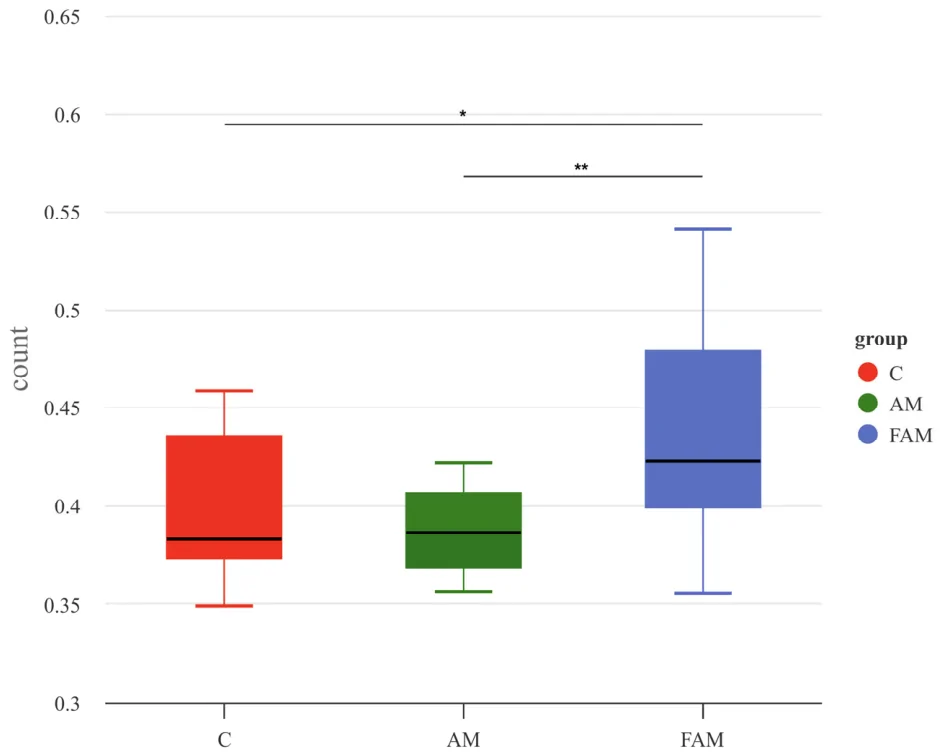
Analysis of the Effects of Rumen Microbial Diversity on Beta Analysis. Note: an asterisk (*) represents a significant difference (0.01 ≤ *p* < 0.05), and double asterisks (**) represent a highly significant difference (0.001 ≤ *p* < 0.01).

As shown in Figure 11, the inter-group difference test of the beta diversity index represents a statistical approach for assessing variations in biodiversity across different groups. Results of beta diversity analysis based on the Kruskal–Wallis test demonstrated significant differences in index distributions among the three groups. Specifically, the median value was highest in the FAM group, followed by the AM group, with the lowest value observed in the Control group. Box plot distributions indicated a wider interquartile range in the FAM group, suggesting greater heterogeneity in ruminal microbial community structure among individual samples. Overall, the beta diversity index in the FAM group was extremely significantly higher than that in the AM group (*p* < 0.01) and significantly higher than that in the Control group (*p* < 0.05), indicating pronounced differences in ruminal microbial community structure among the experimental groups.

##### Effects of AM and FAM on Rumen Microflora of Postpartum Ewes at the Phylum Level

**Figure 12:**
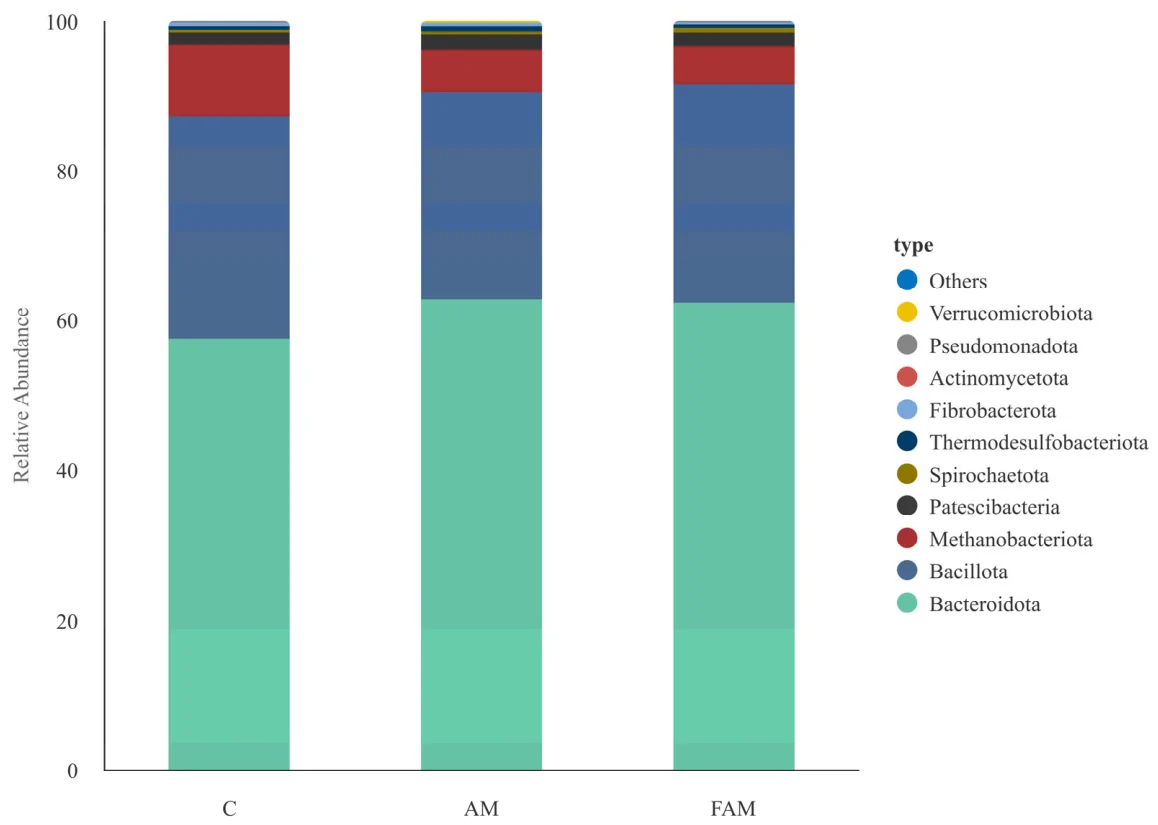
Rumen microbial community composition at phylum level in ewes fed Astragalus and fermented Astragalus.

As shown in Figure 12, rumen microbiota at the phylum level in Control, AM, and FAM were dominated by Bacteroidota (57.55%, 62.80%, 62.27%), Bacillota (29.65%, 27.69%, 29.43%), Methanobacteriota (9.65%, 5.60%, 4.85%), Patescibacteria (1.54%, 2.00%, 1.81%), Spirochaetota (0.54%, 0.52%, 0.65%), Thermodesulfobacteriota (0.46%, 0.62%, 0.49%), Fibrobacterota (0.39%, 0.51%, 0.21%), Actinobacteriota (0.07%, 0.10%, 0.10%), Pseudomonadota (0.04%, 0.05%, 0.07%), and Verrucomicrobiota (0.03%, 0.03%, 0.02%).

##### Effects of AM and FAM on Rumen Microflora of Postpartum Ewes at Genus Level

**Figure 13:**
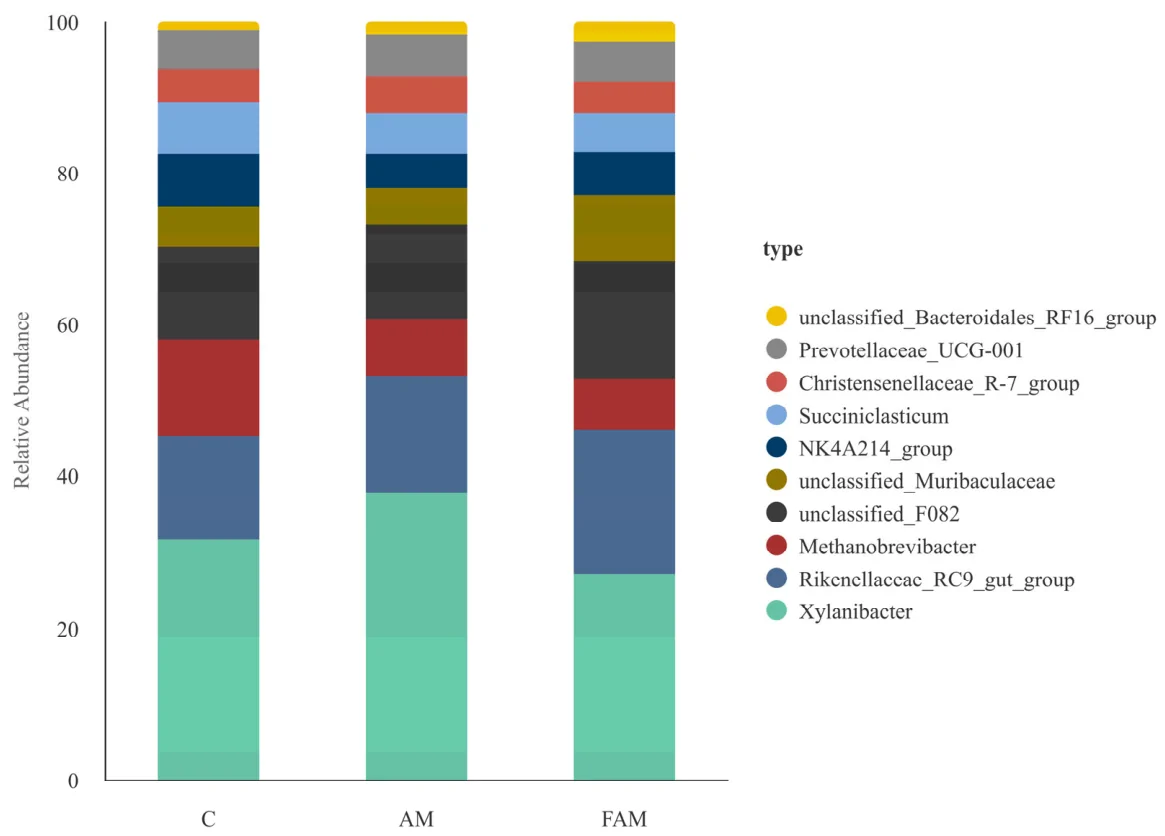
Rumen microbial community composition at genus level in ewes fed Astragalus and fermented Astragalus.

As shown in Figure 13, genus-level rumen microbial composition was characterized in the Control, AM, and FAM groups. The predominant annotated genera were *Xylanibacter* (24.18%, 27.61%, 19.31%), *Rikenellaceae_RC9_gut_group* (10.33%, 11.28%, 13.48%), *Methanobrevibacter* (9.50%, 5.44%, 4.76%), *unclassified_F082* (9.30%, 9.05%, 11.10%), *unclassified_Muribaculaceae* (4.04%, 3.58%, 6.09%), *NK4A214_group* (5.25%, 3.26%, 4.05%), *Succiniclasticum* (5.27%, 3.94%, 3.63%), *Christensenellaceae_R*–*7_group* (3.21%, 3.42%, 2.85%), *Prevotellaceae UCG-001* (3.87%, 4.06%, 3.71%), and *unclassified_Bacteroidales_RF16_group* (0.93%, 1.32%, 2.00%).

##### Rumen Microbiota Structure in AM/FAM-Supplemented Postpartum Ewes

**Figure 14:**
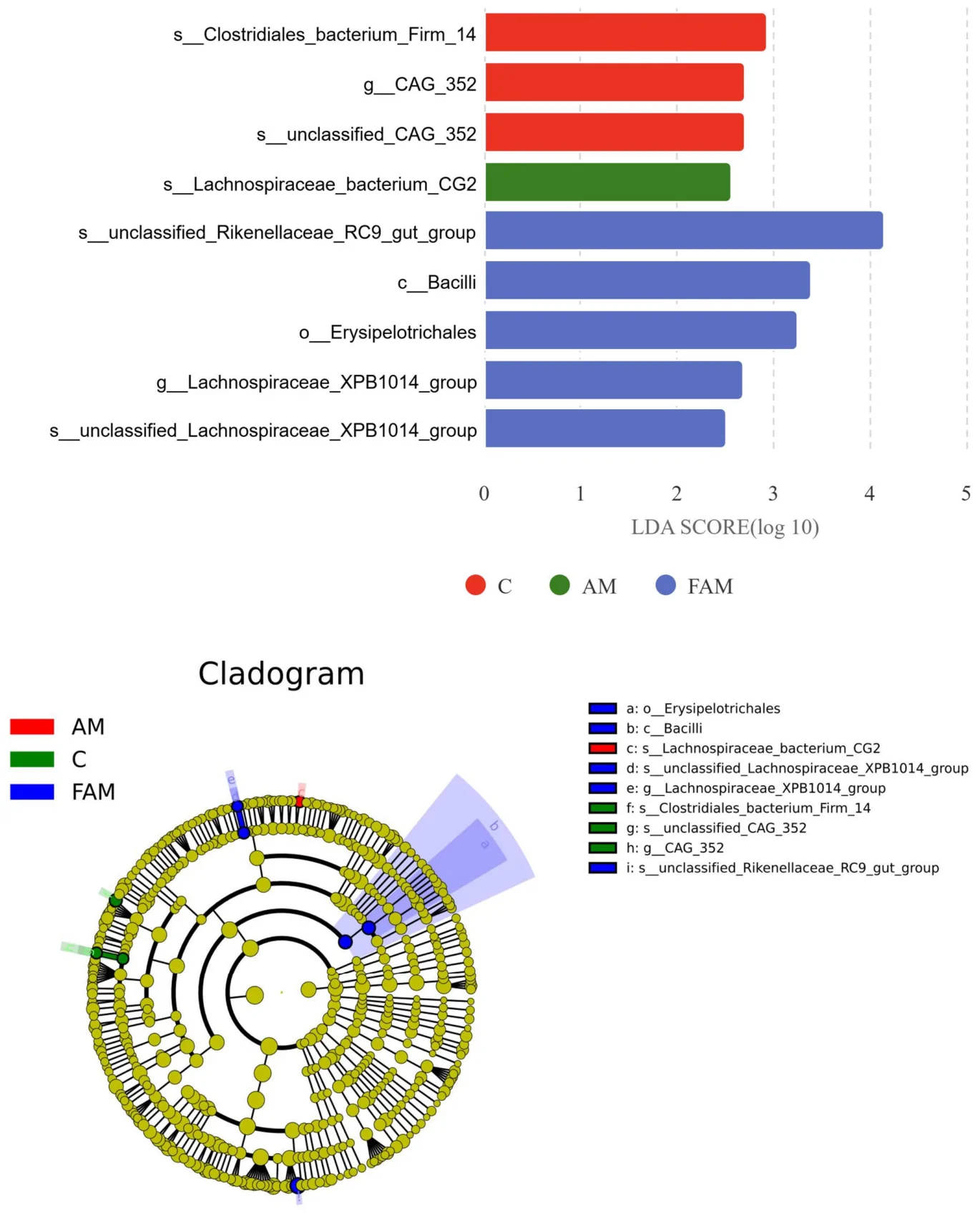
LEfSe analysis of rumen microbiota in ewes supplemented with Astragalus and fermented Astragalus. Note: LEfSe analysis; g: Species phylogenetic tree. The abbreviations c, o, g and s represent the taxonomic levels of class, order, genus and species, respectively.

Integrating the above community-structure and taxonomic-composition analyses indicates that FAM modulated the rumen microbiota of ewes to a certain extent. LEfSe analysis was subsequently conducted using LDA score > 2.5 and *p* < 0.05 as screening criteria to identify taxa with significantly differential relative abundances among the three groups; the results are shown in Figure 14.

In the Control group, relative abundances of *s_Clostridiales_bacterium_Firm_14* (LDA = 2.96), *g_CAG_352* (LDA = 2.72), and *s_unclassified_CAG_352* (LDA = 2.72) were significantly higher than those in the AM and FAM groups.

In the AM group, relative abundance of *s_Lachnospiraceae_bacterium_CG2* (LDA = 2.53) was significantly higher than that in the Control and FAM groups.

In the FAM group, relative abundances of *s_unclassified_Rikenellaceae_RC9_gut_group* (LDA = 4.14), c_Bacilli (LDA = 3.39), o_Erysipelotrichales (LDA = 3.24), *g_Lachnospiraceae_XPB1014_group* (LDA = 2.68), and *s_unclassified_Lachnospiraceae_XPB1014_group* (LDA = 2.49) were significantly higher than those in the Control and AM groups.

### 3.2. Effects of Supplementary Feeding AM and FAM on Lambs

#### 3.2.1. Effects of Supplementary Feeding AM and FAM on Growth Performance of Newborn Lambs

##### Effects of Supplementary Feeding AM and FAM on Body Weight and Average Daily Gain of Newborn Lambs

As shown in Table 5, no significant treatment × time interactions were detected for body weight or ADG in either male or female newborn lambs (*p* > 0.05), whereas time exerted an extremely significant effect (*p* < 0.01). In male lambs, treatment significantly affected ADG (*p* < 0.05), with the FAM group showing markedly greater ADG than the Control and AM groups (*p* < 0.05). Lamb body weight and ADG only changed significantly over time without treatment-time interaction (*p* < 0.01). FAM supplementation notably improved the average daily gain of male lambs, while no treatment differences on body weight were observed across all lambs.

##### Effects of Supplementary Feeding AM and FAM on Growth Axis Hormone Levels of Newborn Lambs

As shown in Table 6, AM and FAM supplementation led to extremely significant increases in plasma GH and IGF-1 concentrations in lambs, reflected in treatment effects, time effects, and treatment × time interactions (*p* < 0.01). Plasma GH and IGF-1 levels were extremely significantly higher in the FAM group than in the AM and Control groups (*p* < 0.01), and values in the AM group were also extremely significantly higher than those in the Control group (*p* < 0.01). Both AM and FAM significantly raised lamb plasma GH and IGF-1, with FAM showing a superior boosting effect. The concentrations of GH and IGF–-1 were co-regulated by treatment, sampling time, and their interaction.

#### 3.2.2. Effects of AM and FAM Supplementation on Plasma Immunoglobulin Contents of Newborn Lambs

As shown in Table 7, FAM supplementation produced extremely significant elevations in plasma IgA and IgG concentrations in lambs across treatment effects, time effects, and treatment × time interactions (*p* < 0.01). Plasma IgA and IgG levels in the FAM group were extremely significantly higher than those in the Control and AM groups (*p* < 0.01), and the AM group also showed extremely significantly higher IgA and IgG than the Control group (*p* < 0.01). No significant between-group difference was observed in plasma IgM concentration (*p* > 0.05). Both AM and FAM treatments markedly increased lamb plasma IgA and IgG, with FAM exhibiting a stronger promoting effect. Neither treatment altered the plasma IgM content of lambs, and dynamic changes of IgA and IgG were jointly affected by treatment and sampling time.

#### 3.2.3. Effects of AM and FAM Supplementation on Plasma Antioxidant Capacity of Newborn Lambs

As shown in Table 8, the T-AOC of newborn lambs exhibited extremely significant differences for time effect and interaction between group and time (*p* < 0.01), while no significant difference was observed in group effect (*p* > 0.05). For CAT, extremely significant differences were detected in group effect, time effect and their interaction (*p* < 0.01); the CAT activities in the Astragalus group and fermented Astragalus group were both extremely higher than those in the control group (*p* < 0.01). Extremely significant differences in SOD and GSH-Px were found for group effect and time effect (*p* < 0.01), whereas their interaction had no significant influence (*p* > 0.05). The SOD activity in the Astragalus group was markedly higher than that in the control group and fermented Astragalus group (*p* < 0.01), and the control group also showed a significantly higher SOD level compared with the fermented Astragalus group (*p* < 0.01). Additionally, the GSH–Px activity in the control group was extremely higher than that in the Astragalus group and fermented Astragalus group (*p* < 0.01). For nitric oxide (NO), extremely significant differences were observed in time effect and group-time interaction (*p* < 0.01), but group effect did not reach a significant level (*p* > 0.05). The MDA presented a significant difference only in time effect (*p* < 0.05), with no significant differences in group effect and group-time interaction (*p* > 0.05). Astragalus and fermented Astragalus supplementation improved neonatal lamb CAT activity, while unfermented Astragalus elevated SOD activity but reduced GSH-Px activity. Time exerted universal effects on all antioxidant indicators, and group × time interactions existed for T-AOC, CAT and NO; MDA levels were only affected by sampling time without treatment-related changes.

### 3.3. Correlation Analysis

#### 3.3.1. Correlation Analysis Between Plasma Differential Metabolites and Differential Rumen Microflora at Genus Level in Ewes

**Figure 15:**
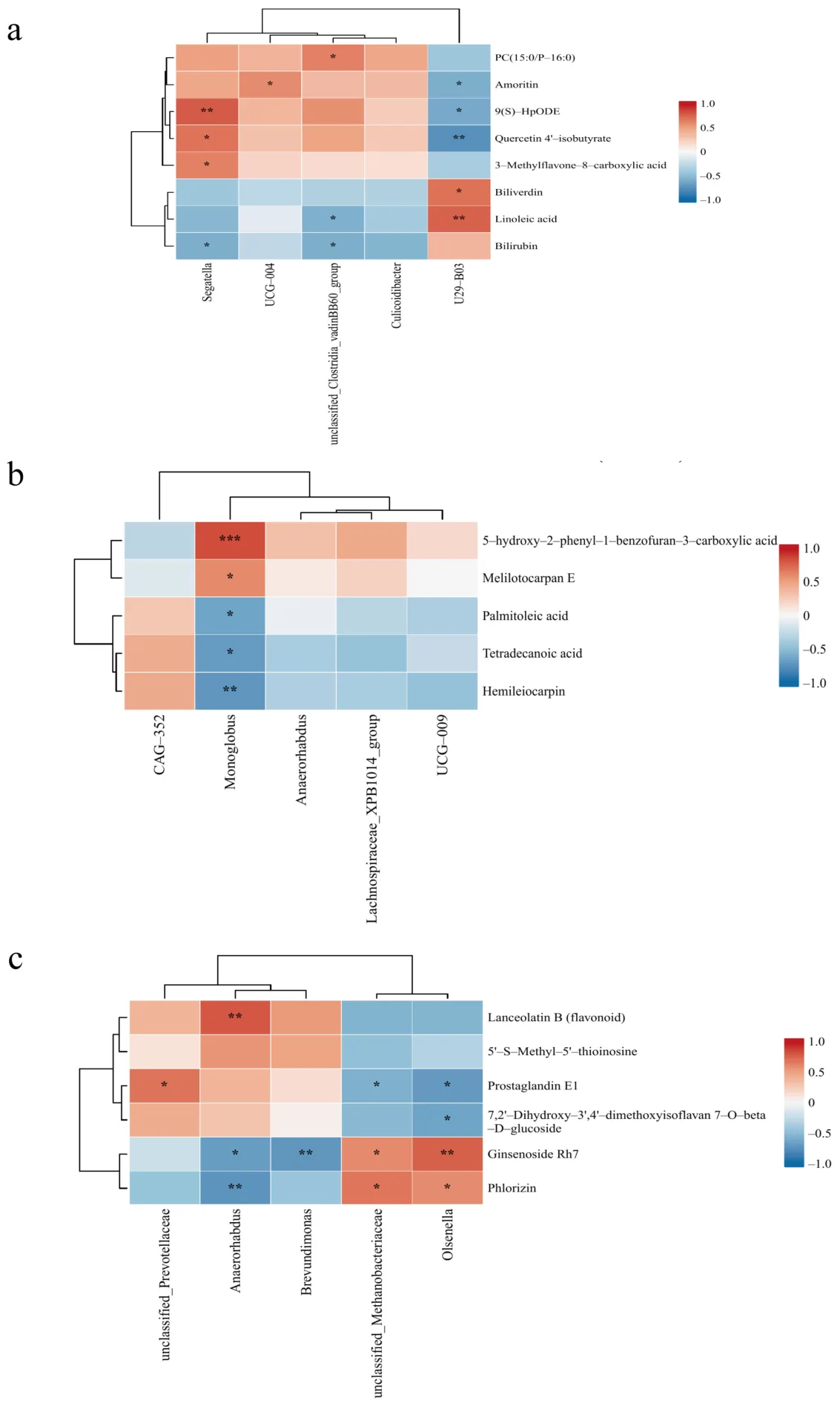
(**a**) Correlation analysis between differentially expressed plasma metabolites and ruminal differential associated microbial communities at the genus level of ewes (AM group vs. Control group); (**b**) Correlation analysis between differentially expressed plasma metabolites and ruminal differential associated microbial communities at the genus level of ewes (FAM group vs. Control group); (**c**) Correlation analysis between differentially expressed plasma metabolites and ruminal differential associated microbial communities at the genus level of ewes (FAM group vs. AM group). Note: an asterisk (*) indicates significant difference (0.01 ≤ *p* < 0.05); double asterisks (**) indicate highly significant difference (0.001 ≤ *p* < 0.01); triple asterisks (***) indicate extremely significant difference (*p* < 0.001).

Differential plasma metabolites across groups, together with dominant microbiota at the family and genus levels, were selected for correlation analysis. Pearson correlation analysis was performed to generate a correlation clustering heatmap (Figure 15).

Comparative analysis between the Control and AM groups revealed distinct correlation patterns, and correlations between differential metabolites and ruminal microorganisms at the genus level are presented in Figure 15a. In the AM group, *Segatella* exhibited an extremely significant positive correlation with 9(S)–HpODE (*p* < 0.01), significant positive correlations with quercetin 4′–isobutyrate and 3-methylflavone-8-carboxylic acid (*p* < 0.05), and a significant negative correlation with bilirubin (*p* < 0.05). *UCG–004* showed a significant positive correlation with amoritin (*p* < 0.05). *The unclassified Clostridia_vadinBB60_group* was positively correlated with PC(15:0/P–16:0) (*p* < 0.05) and negatively correlated with bilirubin and linoleic acid (*p* < 0.05). *U29-B03* exhibited significant negative correlations with amoritin and 9(S)-HpODE (*p* < 0.05), an extremely significant negative correlation with quercetin 4′–isobutyrate (*p* < 0.01), an extremely significant positive correlation with linoleic acid (*p* < 0.01), and a significant positive correlation with biliverdin (*p* < 0.05).

As shown in Figure 15b, comparative analysis of the Control and FAM groups also identified distinct correlation patterns. In the FAM group, *Monoglobus* exhibited an extremely significant positive correlation with 5-hydroxy-2-phenyl-1-benzofuran-3-carboxylic acid (*p* < 0.001), a significant positive correlation with melilotocarpan E (*p* < 0.05), significant negative correlations with tetradecanoic acid and palmitoleic acid (*p* < 0.05), and an extremely significant negative correlation with hemileiocarpin (*p* < 0.01).

As illustrated in Figure 15c, comparative analysis between the AM and FAM groups further revealed distinct correlation patterns. In the FAM group, *unclassified_Prevotellaceae* showed a significant positive correlation with prostaglandin E_1_ (*p* < 0.05). *Anaerorhabdus* exhibited an extremely significant positive correlation with lanceolatin B (flavonoid) (*p* < 0.01), an extremely significant negative correlation with phlorizin (*p* < 0.01), and a significant negative correlation with ginsenoside Rh7 (*p* < 0.05). *Brevundimonas* was extremely significantly negatively correlated with ginsenoside Rh7 (*p* < 0.01). *Unclassified_Methanobacteriaceae* showed a significant negative correlation with prostaglandin E_1_ (*p* < 0.05), together with significant positive correlations with phlorizin and ginsenoside Rh7 (*p* < 0.05). *Olsenella* was significantly negatively correlated with prostaglandin E1 and 7,2′-dihydroxy-3′,4′-dimethoxyisoflavan 7-O-β-D-glucoside (*p* < 0.05), exhibited an extremely significant positive correlation with ginsenoside Rh7 (*p* < 0.01), and showed a significant positive correlation with phlorizin (*p* < 0.05).

#### 3.3.2. Analysis of the Correlation Between Differential Metabolites in Ewe Plasma and Antioxidant Capacity

**Figure 16:**
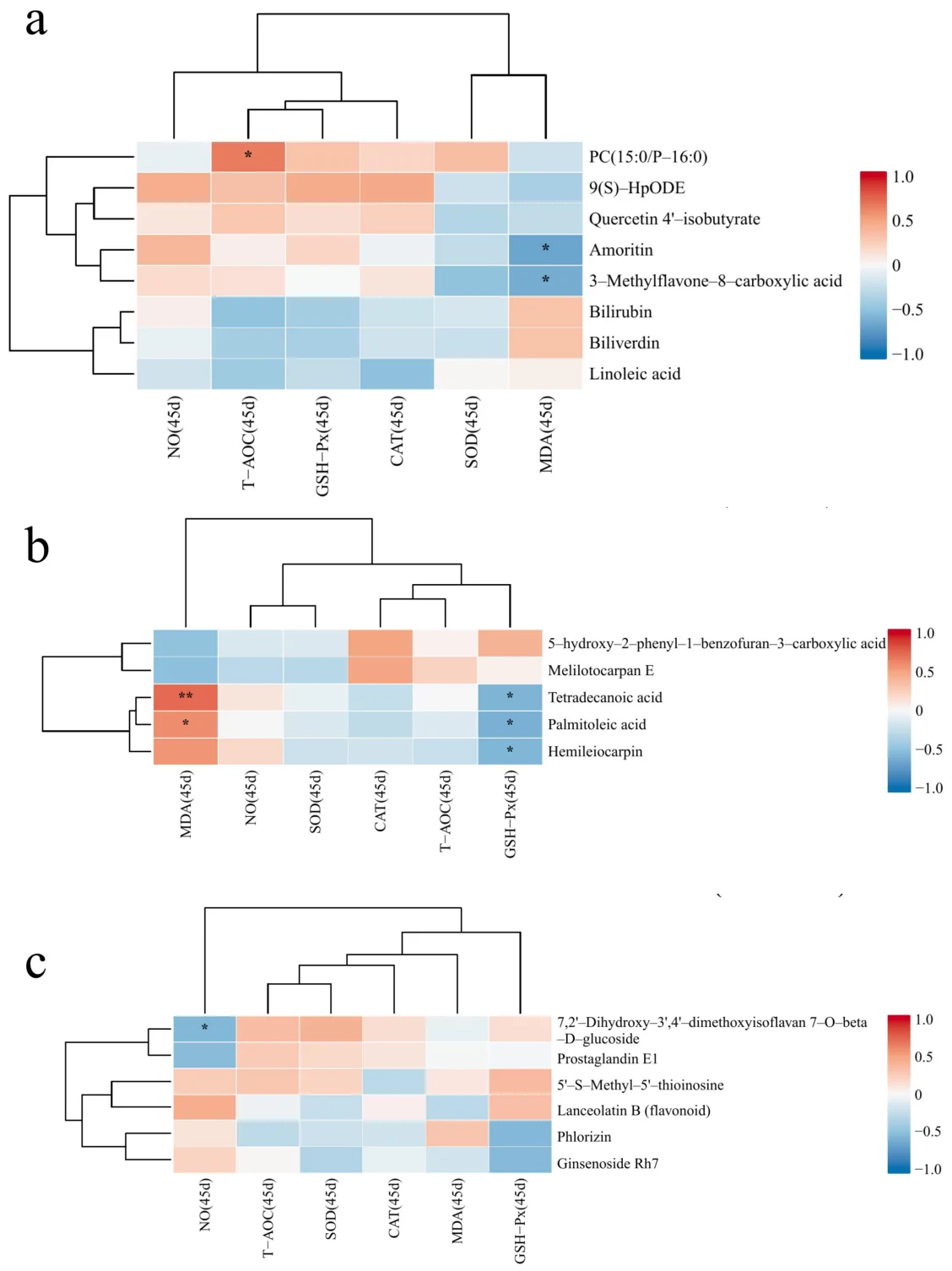
(**a**) Correlation analysis of differentially expressed metabolites in ewe plasma and antioxidant capacity (AM group vs. Control group); (**b**) Correlation analysis of differentially expressed metabolites in ewe plasma and antioxidant capacity (FAM group vs. Control group); (**c**) Correlation analysis of differentially expressed metabolites in ewe plasma and antioxidant capacity (FAM group vs. AM group). Note: an asterisk (*) indicates significant difference (0.01 ≤ *p* < 0.05); double asterisks (**) indicate highly significant difference (0.001 ≤ *p* < 0.01).

In the comparison between the AM group and the Control group, PC (15:0/P-16:0) showed a significant positive correlation with T-AOC (*p* < 0.05); amoritin and 3-methylflavone-8-carboxylic acid exhibited significant negative correlations with MDA (*p* < 0.05) (Figure 16a). MDA was extremely significantly positively correlated with tetradecanoic acid (*p* < 0.01) and significantly positively correlated with palmitoleic acid (*p* < 0.05).

In the comparison between the FAM group and the Control group, tetradecanoic acid, palmitoleic acid and hemileiocarpin were significantly negatively correlated with GSH-Px (*p* < 0.05) (Figure 16b).

In the comparison between the FAM group and the AM group, 7,2′-dihydroxy-3′,4′-dimethoxyisoflavan 7-O-β-D-glucoside displayed a significant negative correlation with NO (*p* < 0.05) (Figure 16c).

#### 3.3.3. Correlation Analysis of Differential Abundance Microbiota in Ewe Rumen with Millk Composition

**Figure 17:**
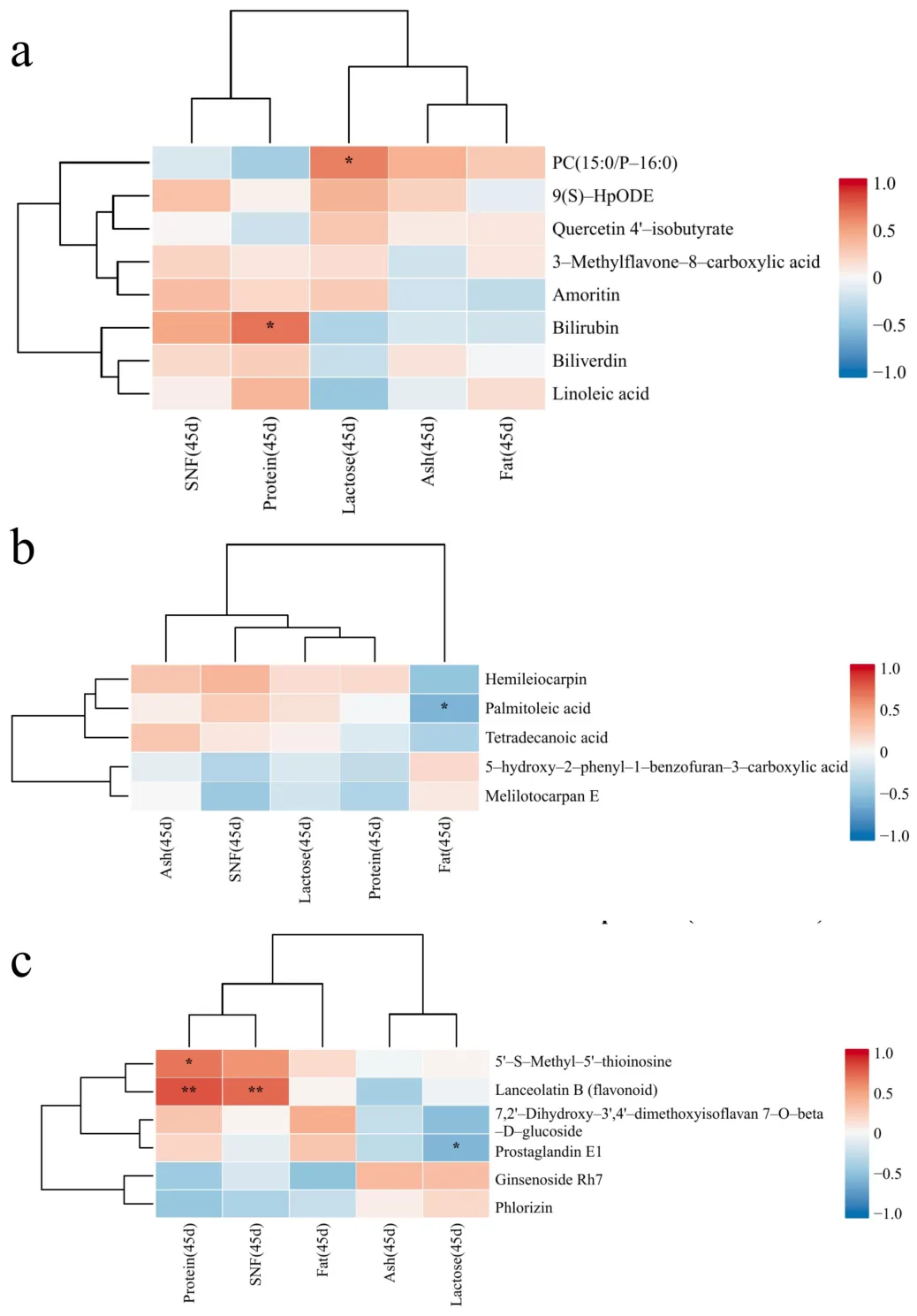
(**a**) Correlation analysis of differentially expressed metabolites in ewe plasma and milk composition (AM group vs. Control group); (**b**) Correlation analysis of differentially expressed metabolites in ewe plasma and milk composition (FAM group vs. Control group); (**c**) Correlation analysis of differentially expressed metabolites in ewe plasma and milk composition (FAM group vs. AM group). Note: an asterisk (*) indicates significant difference (0.01 ≤ *p* < 0.05); double asterisks (**) indicate highly significant difference (0.001 ≤ *p* < 0.01).

In the comparison between the AM group and the Control group, phosphatidylcholine (15:0/P-16:0) (PC(15:0/P-16:0)) had a significant positive correlation with lactose (*p* < 0.05), and bilirubin was significantly positively correlated with protein (*p* < 0.05) (Figure 17a).

In the comparison between the FAM group and the Control group, palmitoleic acid showed a significant negative correlation with fat (*p* < 0.05) (Figure 17b).

In the comparison between the FAM group and the AM group, lanceolatin B (flavonoid) exhibited an extremely significant positive correlation with protein and SNF (*p* < 0.01); 5′-S-methyl-5′-thioinosine was significantly positively correlated with protein (*p* < 0.05), while prostaglandin E1 had a significant negative correlation with lactose (*p* < 0.05) (Figure 17c).

#### 3.3.4. Analysis of the Relationship Between Rumen Microorganisms (At the Genus Level) and Plasma Antioxidant Capacity in Ewes

**Figure 18:**
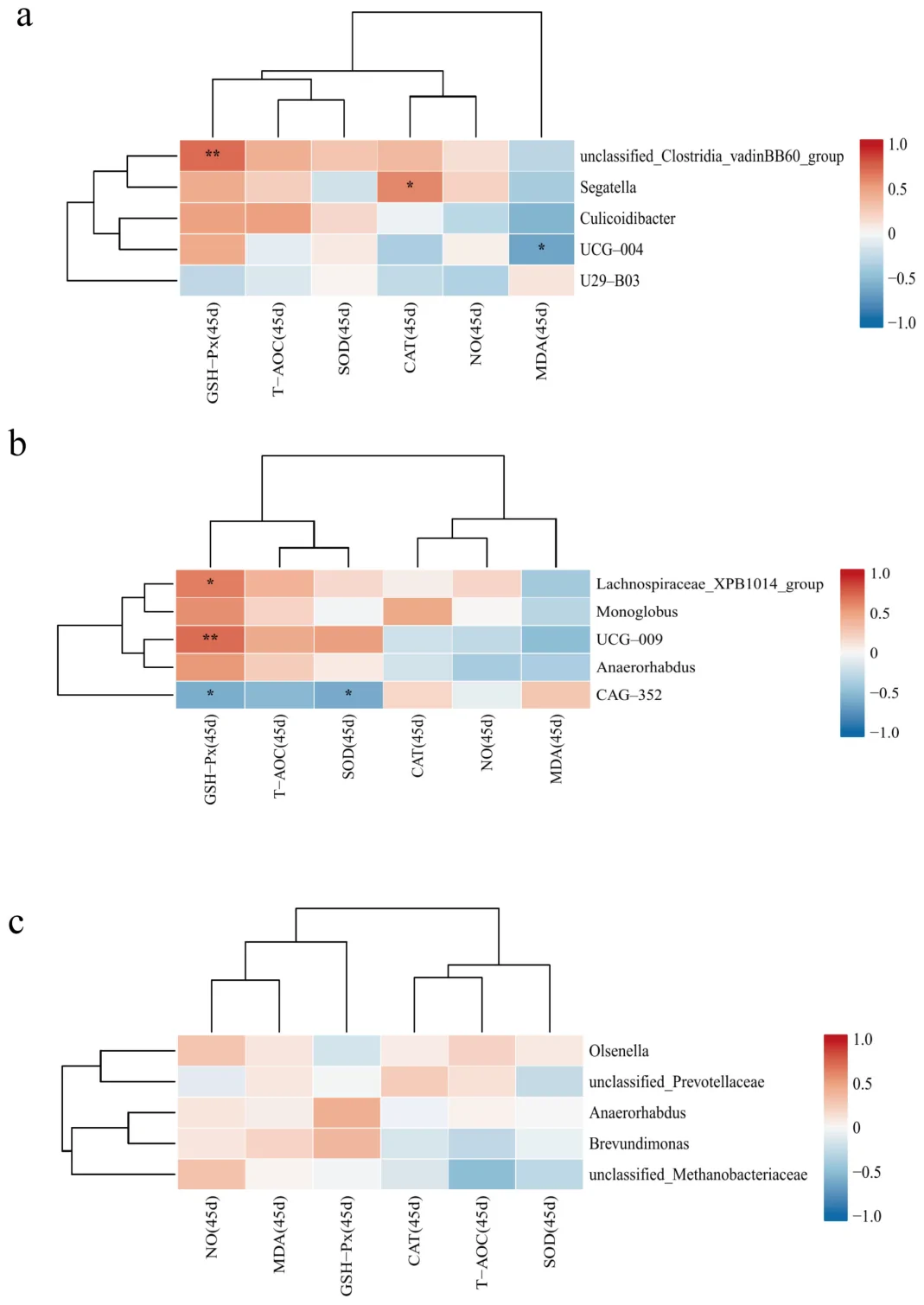
(**a**) Correlation analysis of ewe ruminal microbial communities at the genus level and antioxidant capacity (AM group vs. Control group); (**b**) Correlation analysis of ewe ruminal microbial communities at the genus level and antioxidant capacity (FAM group vs. Control group); (**c**) Correlation analysis of ewe ruminal microbial communities at the genus level and antioxidant capacity (FAM group vs. AM group). Note: an asterisk (*) indicates significant difference (0.01 ≤ *p* < 0.05); double asterisks (**) indicate highly significant difference (0.001 ≤ *p* < 0.01).

Compared with the Control group, in the AM group, *unclassified Clostridia_vadinBB60_group* was extremely significantly positively correlated with GSH-Px (*p* < 0.01), Segatella was significantly positively correlated with CAT (*p* < 0.05), and *UCG-004* was significantly negatively correlated with MDA (*p* < 0.05) (Figure 18a). For the FAM group relative to the Control group, *UCG-009* was extremely significantly positively correlated with GSH-Px (*p* < 0.01), *CAG-352* was significantly negatively correlated with GSH-Px and SOD (*p* < 0.05), and *Lachnospiraceae_XPB1014_group* was significantly positively correlated with GSH-Px (*p* < 0.05) (Figure 18b).

#### 3.3.5. Analysis of the Relationship Between Rumen Microorganisms (At the Genus Level) and Milk Composition in Ewes

**Figure 19:**
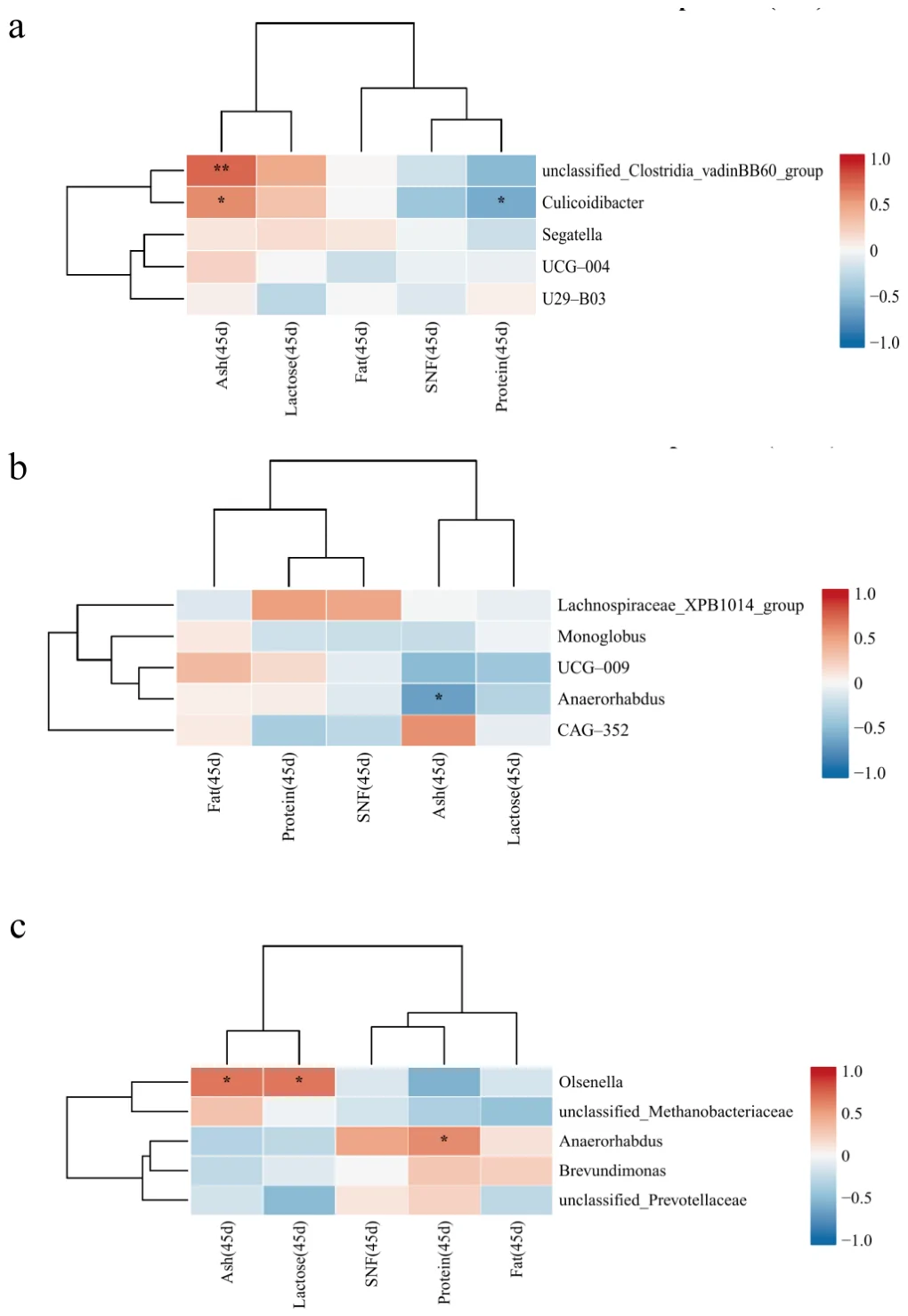
(**a**) Correlation analysis of rumen microbiota at the genus level in ewes and milk composition (AM group vs. Control group); (**b**) Correlation analysis of rumen microbiota at the genus level in ewes and milk composition (FAM group vs. Control group); (**c**) Correlation analysis of rumen microbiota at the genus level in ewes and milk composition (FAM group vs. AM group). Note: an asterisk (*) indicates significant difference (0.01 ≤ *p* < 0.05); double asterisks (**) indicate highly significant difference (0.001 ≤ *p* < 0.01).

In the comparison of the AM group with the Control group, *unclassified Clostridia_vadinBB60_group* showed a highly significant positive correlation with Ash (*p* < 0.01), while *Culicoidibacter* exhibited a significant positive correlation with Ash (*p* < 0.05) and a significant negative correlation with Protein (*p* < 0.05) (Figure 19a). In the FAM group versus the Control group, *Anaerorhabdus* was significantly negatively correlated with Ash (*p* < 0.05) (Figure 19b). In the comparison between the FAM group and the AM group, *Anaerorhabdus* was significantly positively correlated with Protein (*p* < 0.05), and *Olsenella* showed significant positive correlations with Ash and Lactose (*p* < 0.05) (Figure 19c).

## 4. Discussion

Highly diverse and complex microbial assemblages—including bacteria, fungi, archaea, and protozoa—reside within the rumen, which serves as the primary digestive organ in ruminant species. This ecosystem plays a central role in cellulose degradation and nutrient biotransformation [20]. Adjustments in dietary structure and shifts in physiological status can substantially modify ruminal function and microbial community composition [21]. In the present study, alpha–diversity indices, including species richness, community diversity, and coverage, were significantly higher in sheep receiving fermented astragalus than in the control group, whereas no significant differences were detected between the raw astragalus group and the control group. Beta-diversity analysis further showed that the diversity index in the FAM group was extremely significantly higher than that in the AM group and significantly higher than that in the Control group. These findings indicate that mixed fermentation with *Bacillus subtilis* and *Bacillus licheniformis* markedly enhanced the modulatory effect of astragalus on ruminal microbial community structure. This effect may be attributable to reduced molecular weight of astragalus polysaccharides (APS) and increased release of bioactive constituents during fermentation. Firmicutes and Bacteroidetes are recognized as the dominant phyla in the rumen of ruminants [22]. Firmicutes are primarily involved in degradation of structural carbohydrates such as fiber and include key fibrolytic taxa, including *Butyrivibrio*, *Pseudobutyrivibrio*, and *Ruminococcus*. Bacteroidetes are closely associated with decomposition of polysaccharides such as starch and are mainly represented by *Bacteroides*, *Prevotella*, and *Rikenella* [23,24]. The persistence of Firmicutes and Bacteroidetes as the dominant phyla following APS supplementation has also been documented in Hu sheep by Ran et al. consistent with our observations. In the present study, Bacteroidetes and Firmicutes were also identified as dominant phyla in both the AM and FAM groups [25]. Moreover, Bacteroidetes abundance exceeded that in the control group, suggesting that APS modulated ruminal microbial structure and provided high-quality carbon substrates for Bacteroidetes proliferation. At the genus level, relative abundances of *Xylanibacter* and *Rikenellaceae_RC9_gut_group* were higher in the AM and FAM groups than in the Control group. As a key fibrolytic genus, *Xylanibacter* secretes cellulolytic enzymes such as xylanase, enabling degradation of complex carbohydrates, including xylan and cellulose, into low-molecular-weight compounds (e.g., oligosaccharides and monosaccharides) utilizable by rumen microorganisms, thereby supporting host energy and nutrient supply [26]. Bioactive constituents in raw and fermented astragalus, including polysaccharides, saponins, and flavonoids, may establish a favorable nutritional milieu for Xylanibacter growth and proliferation, thereby enhancing ruminal fiber-degradation capacity. *Rikenellaceae_RC9_gut_group* is mainly involved in fermentation and utilization of ruminal carbohydrates and improves energetic efficiency, with important implications for maintenance of physiological metabolism and lactation performance in lactating ewes [27]. In addition, shifts in the abundance of these genera may influence the structure and function of related taxa through maintenance of ruminal ecological homeostasis, thereby jointly regulating the ruminal fermentation process in ewes.

Untargeted metabolomic analysis revealed that supplementation with AM and FAM significantly altered the expression profiles of numerous plasma metabolites in ewes. Subsequent screening and KEGG pathway enrichment analysis demonstrated that the differential plasma metabolites were predominantly enriched in linoleic acid metabolism and porphyrin metabolism following astragalus supplementation. Notably, the levels of bilirubin and lumirubin, both involved in porphyrin metabolism, were markedly downregulated. Previous studies have shown that astragalus injection effectively reduces total bilirubin (TBIL) levels in patients with hyperbilirubinemia [28]. Furthermore, four weeks of combined treatment with Astragalus Qi-tonifying decoction and reduced glutathione significantly decreased serum TBIL levels, improved liver function, and alleviated oxidative stress and inflammatory responses in patients with liver cancer following interventional therapy [29], consistent with the present findings. The level of 9(S)–HpODE, a metabolite involved in linoleic acid metabolism, was significantly increased. Linoleic acid can be catalyzed by lipoxygenase to generate 9(S)–HpODE, and its pronounced upregulation suggested activation of the linoleic acid metabolic pathway by astragalus supplementation, thereby accelerating the oxidative degradation of linoleic acid, consistent with the significant reduction in linoleic acid content. In addition, flavonoid bioactive compounds in astragalus, including quercetin 4′-isobutyrate, amoritin, and 3-methylflavone–8–carboxylic acid, were all markedly upregulated. These findings further indirectly support that dietary astragalus supplementation activated linoleic acid metabolism and promoted linoleic acid degradation in ewes.

Differential plasma metabolites induced by fermented astragalus supplementation were predominantly enriched in the fatty acid biosynthesis pathway. Tetradecanoic acid and palmitoleic acid serve as key intermediate metabolites in lipid synthesis and desaturation pathways. As a saturated fatty acid, tetradecanoic acid is not only a direct product of fatty acid biosynthesis but also an essential substrate for carbon chain elongation and desaturation reactions [30]. Palmitoleic acid biosynthesis is highly dependent on the activity of stearoyl-CoA desaturase (SCD), which catalyzes the conversion of saturated fatty acids into monounsaturated fatty acids [31]. Following fermented astragalus (FAM) supplementation, the levels of tetradecanoic acid and palmitoleic acid were significantly reduced. Short-chain fatty acids, such as butyric acid, together with flavonoid metabolites generated during FAM fermentation, may activate AMPK, thereby suppressing fatty acid synthase (FASN) and stearoyl–CoA desaturase 1 (SCD1) activity and reducing palmitoleic acid biosynthesis. Activation of AMPK suppresses downstream FASN and SCD1 activity, directly inhibiting the de novo synthesis of tetradecanoic acid and palmitoleic acid [32]. Concurrently, AMPK activation enhances carnitine palmitoyltransferase (CPT) activity, promoting the mitochondrial transport of fatty acids, including C14:0 and C16:1, for β-oxidation and accelerating their catabolism [33]. In addition, flavonoids and isoflavonoids, including melilotocarpan E and 5–hydroxy–2–phenyl–1–benzofuran–3–carboxylic acid, were markedly upregulated. These findings further suggest that FAM modulated plasma metabolism by increasing the abundance of related bioactive compounds, although the underlying mechanisms require further investigation.

Active constituents of astragalus, including astragalosides and polysaccharides, play critical roles in enhancing antioxidant capacity and immune function. Astragaloside IV has been shown to stimulate the proliferation and activity of T lymphocytes, B lymphocytes, macrophages, and neutrophils through multiple pathways, while promoting the development of immune organs [34]. APS can alleviate oxidative stress and improve immune status [35,36] by increasing SOD and GSH–Px activities and reducing MDA content, thereby mitigating reactive oxygen species-induced damage [37]. In addition, astragalosides markedly increase serum GSH–Px activity and decrease MDA levels, consequently enhancing the antioxidant capacity of calves subjected to weaning stress [38]. The present findings are in agreement with Wang, who reported that escalating dietary astragaloside levels significantly enhanced CAT, GSH–Px, and SOD activities while markedly suppressing MDA production [39]. In the current experiment, plasma CAT, SOD, and GSH–Px activities were elevated, whereas MDA and NO contents exhibited decreasing trends in ewes supplemented with raw and fermented astragalus. A previous study by Cao et al. demonstrated that dietary fermented astragalus supplementation effectively improved immune function, growth performance, and digestive capacity in Sichuan white geese [40]. Under the experimental conditions of the present study, fermented astragalus exerted a greater effect on improving the antioxidant capacity of ewes than raw astragalus. Xu and Wang reported that mixed-strain fermentation significantly increased the total saponin content of astragalus residue by 132.06% [41]. Accordingly, the biological activities of astragalosides and polysaccharides were presumed to be enhanced in fermented astragalus relative to the unfermented form. Enhanced antioxidant, anti-inflammatory, and disease-resistance capacities improved the physiological metabolic status of ewes, thereby providing a solid basis for normal lactational performance [42]. Previous studies have confirmed that APS alleviate oxidative stress-induced damage in mammary gland cells, preserve the structural and functional integrity of mammary tissue, and maintain normal lactational activity [43]. Simultaneously, the enhanced anti-inflammatory and disease-resistance effects of astragalosides may reduce the risk of pathogen infection in ewes, thereby preventing declines in milk yield and abnormalities in milk composition [6]. In the present study, dietary supplementation with fermented astragalus markedly increased the total milk yield of ewes. Concurrently, plasma IgA and IgG concentrations, as well as CAT activity, were significantly elevated in lambs, with more pronounced effects observed in the fermented astragalus group. Mixed fermentation of astragalus by Bacillus subtilis and Bacillus licheniformis was presumed to optimize the phospholipid composition and oxidative stability of mammary epithelial cell membrane lipids in ewes, thereby enabling milk fat to function as an efficient carrier of immune-related factors. Moreover, fermentation effectively enhanced the biological activities of APS, astragalosides, and flavonoids. As breast milk constitutes the sole source of passive immunity for neonatal lambs, improved immune function and antioxidant capacity could consequently be acquired through maternal milk intake. The immune system of neonatal lambs remains immature, accompanied by a weak antioxidant defense system, rendering them highly susceptible to pathogenic microorganisms and oxidative stress [44]. Growth and development in lambs are closely associated with maternal nutritional status [45]. Essential nutrients required for postnatal growth are primarily obtained from breast milk. In the present study, supplementation with both raw and fermented astragalus improved lamb growth performance, while serum GH and IGF-1 levels were extremely significantly elevated in both treatment groups. GH promotes protein synthesis and supports the growth and development of bone, muscle, and visceral organs [46]. IGF-1 is secreted in response to GH receptor activation and plays a critical role in early growth, organ maturation, and maintenance of nitrogen metabolic balance in young animals. GH and IGF-1 act synergistically to accelerate protein anabolism and promote skeletal and muscular development in lambs [47,48]. The superior efficacy of fermented astragalus may be attributable to its ability to optimize intestinal microecology and enhance nutrient absorption and utilization, thereby providing an adequate material basis for normal GH/IGF-1 axis function.

A Pearson correlation analysis revealed that the unclassified Clostridia (vadinBB60_group) was simultaneously associated with PC(15:0/P-16:0), bilirubin and linoleic acid, as well as the key antioxidant enzyme (GSH-Px) and milk mineral deposition (Ash). Its negative correlation with linoleic acid suggests a potential involvement in ruminal hydrogenation, while its positive correlation with GSH-Px implies a possible influence on the host’s antioxidant status via selenium metabolism or glutathione regeneration pathways, making it a core potential driver for improving milk quality in the AM group. However, specific functional validation requires further experiments, such as isolation and culture and metagenomic analysis. This study only established the potential association between this bacterium and milk composition and antioxidant indicators at the correlational level, providing a direction for subsequent exploration of the interaction mechanisms between microorganisms and the host. Based on the overall results, adding Astragalus to the basal diet can improve the antioxidant capacity of ewes by regulating the composition of their rumen microbiota, thereby optimizing the nutritional composition of colostrum and providing more adequate nutritional support for the growth and development of lambs. This is further corroborated by the findings in this study showing improved growth performance and elevated levels of growth-related hormones in lambs following Astragalus supplementation, indicating that a mechanism exists whereby maternal nutritional regulation improves offspring growth by influencing lactation performance. Fermented Astragalus demonstrated superior regulatory effects, likely due to the fermentation process reducing the content of antinutritional factors in Astragalus and enhancing the utilization rate of active compounds such as Astragalus polysaccharides. This further corroborates the conclusion that fermentation can enhance the efficacy of Astragalus, consistent with previous studies conducted on other animal species.

## 5. Conclusions

**Figure 20:**
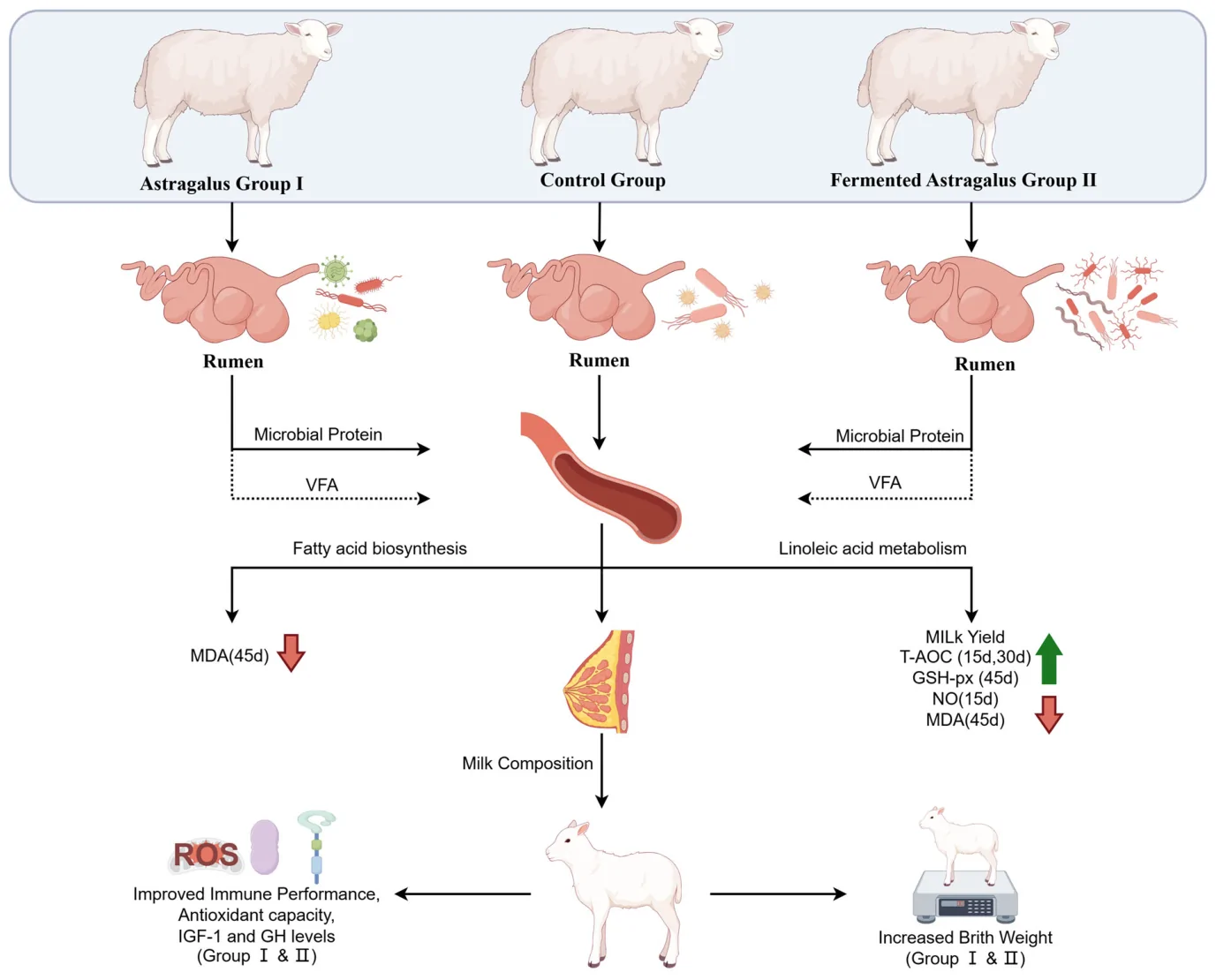
Effects of Fermented Astragalus Supplementation in Ewes on Rumen Function, Antioxidant Status, and Offspring Performance.

Astragalus supplementation increased the abundance of *Xylanibacter* and alleviated oxidative stress in ewes by reducing metabolites such as bilirubin through modulation of porphyrin metabolism. Fermented astragalus primarily increased the abundances of *Rikenellaceae_RC9_gut_group* and *Ruminococcus*, regulated fatty acid biosynthesis, reduced tetradecanoic acid and palmitoleic acid levels, and increased flavonoid contents, thereby further enhancing antioxidant capacity. In addition, fermented astragalus improved milk yield and milk quality. Lambs consequently acquired enhanced immune function and antioxidant capacity through breast milk intake, thereby promoting growth and development. Collectively, fermented astragalus exerted superior effects compared with raw astragalus (Figure 20).

### Limitations

Several limitations of this trial should be stated. Firstly, the number of biological replicates in each group was relatively small, which may weaken the statistical reliability and generalizability of the experimental results. Larger sample sizes are needed in subsequent trials to further verify the conclusions. Secondly, 16S rRNA sequencing was applied to characterize rumen microbial composition and screen differential bacterial taxa, whereas further in-depth bioinformatic analyses were not performed, including microbial functional prediction, species co-occurrence network construction, and integrated correlation analysis between differential bacteria, plasma metabolites and host physiological indicators. These supplementary analyses would help reveal the core functional flora responsible for the regulatory effects of Astragalus and fermented Astragalus. Thirdly, the feeding period of this experiment was limited to 45 days after lambing; long-term feeding trials covering the entire lactation stage are required to explore the sustained regulatory effects of the two herbal additives on rumen microbiota and maternal-lamb production performance.

## Figures and Tables

**Table 1:** Diet Formula Composition and Nutrient Level (dry matter basis) (%).

Ingredients	Content	Nutrient Levels ^2^	Content
Whole corn silage	35.83	CP	14.60
Corn	19.84	EE	2.82
Wheat bran	8.56	Ash	6.82
Soybean meal	12.0	NDF	36.21
Cotton seed meal	4.2	ADF	20.5
Sorghum stalks	7.54	Ca	0.52
Bioactive peptide	6.21	P	0.38
NaHCO_3_	0.51	ME/(MJ·kg^−1^)	10.13
NaCl	0.31		
Premix ^1^	5		
Total	100		

Notes: ^1^ Per kilogram of premix contains: vitamin A 150,000 IU, vitamin D_3_ 8000 IU, vitamin E 1000 IU, vitamin K_3_ 10 mg, copper 150 mg, zinc 600 mg, manganese 450 mg, iron 600 mg, iodine 15 mg, cobalt 5 mg, selenium 5 mg. ^2^ All nutritional levels are determined values, and metabolizable energy is a calculated value.

**Table 2:** Effects of AM and FAM Supplementation on Milk Components of Postpartum Ewes (*n* = 6).

Items	Groups			SEM	*p*-Value		
	Control Group	AM Group	FAM Group		Group	Time	Group × Time
Fat (%)	8.33 ^B^	8.43 ^B^	9.20 ^A^	0.17	<0.01	<0.01	0.26
Protein (%)	7.89	7.72	7.98	0.11	0.31	<0.01	0.62
Lactose (%)	3.93	3.99	3.88	0.05	0.28	<0.01	0.41
Ash (%)	1.04	1.00	1.07	0.05	0.55	<0.01	0.09
SNF (%)	12.78	12.55	12.58	0.18	0.61	<0.01	0.92

Note: Separate uppercase tags mean highly notable differences (*p* < 0.01). Uniform or missing tags correspond to non-divergent results (*p* > 0.05).

**Table 3:** Effects of Dietary Supplementation with Astragalus and Fermented Astragalus on Plasma Antioxidant Capacity in Postpartum Ewes (*n* = 6).

Items	Groups			SEM	*p*-Value		
	Control Group	AM Group	FAM Group		Group	Time	Group × Time
T-AOC (U/mL)	8.25 ^B^	8.22 ^B^	8.54 ^A^	0.10	<0.01	<0.01	<0.01
CAT (U/mL)	6.98	7.08	7.37	0.20	0.46	<0.01	0.79
SOD (U/mL)	8.50	8.65	8.69	0.20	0.68	<0.01	1.00
GSH-Px (U/mL)	101.38 ^b^	105.50 ^ab^	107.80 ^a^	1.66	0.02	<0.01	0.89
NO (nmol/mL)	341.99 ^B^	347.68 ^A^	331.65 ^C^	2.72	<0.01	<0.01	0.24
MDA (nmol/mL)	1.28 ^A^	1.19 ^AB^	1.03 ^B^	0.06	0.01	<0.01	0.06

Note: Unique lowercase tags on matching data reflect minor statistical gaps (*p* < 0.05); separate uppercase tags mean highly notable differences (*p* < 0.01). Uniform or missing tags correspond to non-divergent results (*p* > 0.05).

**Table 4:** Analysis of Rumen Microbiota Alpha-diversity in Postpartum Ewes Supplemented with Astragalus or Fermented Astragalus (*n* = 6).

Items	Groups			*p*-Value
	Con Group	AM Group	FAM Group	
chao1	1457.30 ± 154.43 ^B^	1555.38 ± 137.37 ^B^	2187.27 ± 360.09 ^A^	<0.01
observed_features	1453.83 ± 155.15 ^B^	1552.00 ± 136.55 ^B^	2144.17 ± 341.82 ^A^	<0.01
simpson	0.99 ± 0.01	1.00 ± 0.00	1.00 ± 0.00	0.28
shannon	9.27 ± 0.41 ^b^	9.59 ± 0.15 ^ab^	9.90 ± 0.47 ^a^	0.03
dominance	0.01 ± 0.01	0.01 ± 0.01	0.01 ± 0.01	0.28
goods_coverage	1.00 ± 0.00 ^A^	1.00 ± 0.00 ^A^	0.99 ± 0.00 ^B^	<0.01
pielou_e	0.88 ± 0.03	0.91 ± 0.01	0.90 ± 0.02	0.24

Note: Unique lowercase tags on matching data reflect minor statistical gaps (*p* < 0.05); separate uppercase tags mean highly notable differences (*p* < 0.01). Uniform or missing tags correspond to non-divergent results (*p* > 0.05).

**Table 5:** Effects of Astragalus and Fermented Astragalus on Body Weight of Newborn Lambs.

Items	Groups			SEM	*p*-Value		
	Control Group	AM Group	FAM Group		Group	Time	Group × Time
Body Weight of Male Lambs (n = 8)/kg	9.59	9.62	10.83	0.36	0.07	<0.01	0.12
Average Daily Gain of Male Lambs (n = 7)/kg	0.20 ^b^	0.20 ^b^	0.25 ^a^	0.02	0.04	<0.01	0.63
Body Weight of Female Lambs (n = 7)/kg	9.07	9.65	9.62	0.29	0.29	<0.01	0.87
Average Daily Gain of Female Lambs (n = 8)/kg	0.20	0.21	0.21	0.02	0.65	<0.01	0.79

Note: Unique lowercase tags on matching data reflect minor statistical gaps (*p* < 0.05). Uniform or missing tags correspond to non-divergent results (*p* > 0.05).

**Table 6:** Effects of supplemental Astragalus and fermented Astragalus on growth hormone levels in newborn lambs (*n* = 6).

Items	Groups			SEM	*p*-Value		
	Control Group	AM Group	FAM Group		Group	Time	Group × Time
GH (pg/mL)	99.13 ^C^	100.73 ^B^	116.30 ^A^	1.72	<0.01	<0.01	<0.01
IGF-1 (ng/mL)	2.09 ^C^	2.74 ^B^	3.88 ^A^	0.10	<0.01	<0.01	<0.01

Note: Separate uppercase tags mean highly notable differences (*p* < 0.01). Uniform or missing tags correspond to non-divergent results (*p* > 0.05).

**Table 7:** Globulin Levels in Lamb’s Plasma following AM and FAM Supplementation (*n* = 6, g/L).

Items	Groups			SEM	*p*-Value		
	Control Group	AM Group	FAM Group		Group	Time	Group × Time
IgA (g/L)	3.60 ^C^	3.88 ^B^	4.20 ^A^	0.10	<0.01	<0.01	<0.01
IgG (g/L)	12.42 ^C^	13.51 ^B^	15.43 ^A^	0.55	<0.01	<0.01	<0.01
IgM (g/L)	3.92	3.89	3.66	0.15	0.43	0.05	0.08

Note:Separate uppercase tags mean highly notable differences (*p* < 0.01). Uniform or missing tags correspond to non-divergent results (*p* > 0.05).

**Table 8:** Effects of Astragalus and Fermented Astragalus on Antioxidant Capacity in Plasma of Newborn Lambs (*n* = 6).

Items	Groups			SEM	*p*-Value		
	Control Group	AM Group	FAM Group		Group	Time	Group × Time
T-AOC (U/mL)	9.80	9.69	9.64	0.10	0.41	<0.01	<0.01
CAT (U/mL)	4.94 ^C^	6.32 ^A^	6.18 ^B^	0.28	<0.01	<0.01	<0.01
SOD (U/mL)	12.62 ^B^	13.71 ^A^	11.80 ^C^	0.47	<0.01	<0.01	0.07
GSH–Px (U/mL)	40.30 ^A^	37.85 ^B^	37.55 ^B^	1.36	0.01	<0.01	0.71
NO (nmol/mL)	160.20	160.08	166.48	2.72	0.05	<0.01	<0.01
MDA (nmol/mL)	1.01	1.04	1.09	0.06	0.58	0.03	0.19

Note: Separate uppercase tags mean highly notable differences (*p* < 0.01). Uniform or missing tags correspond to non-divergent results (*p* > 0.05).

## Data Availability

Public online databases store all original omics data acquired throughout the present experiment. For 16S rRNA sequencing datasets, readers can retrieve the complete sequence information at NCBI SRA through the link https://www.ncbi.nlm.nih.gov/sra/PRJNA1494529 (accessed on 27 July 2026), and detailed repository and accession information is provided in the main text. Additionally, raw untargeted metabolomics data together with their supporting annotation files are preserved in the OMIX BioProject repository, bearing the BioProject identifier PRJCA069704 (accessed on 13 July 2026) and submission ID subPRO101362.

## Abbreviations

ADF	Acid Detergent Fiber
AM	Astragalus membranaceus
ANOVA	One–way analysis of variance
APS	Astragalus Polysaccharides
BW	Body Weight
CP	Crude Protein
CAT	Catalase
Ca	Calcium
DM	Dry Matter
EE	Ether Extract
GH	Growth Hormone
GSH-Px	Glutathione Peroxidase
IGF-1	Insulin–like Growth Factor–1
IgA	Immunoglobulin A
IgM	Immunoglobulin M
IgG	Immunoglobulin G
MDA	Malondialdehyde
NO	Nitric Oxide
NDF	Neutral detergent fiber
P	Phosphorus
SOD	Superoxide Dismutase
SNF	Solids-Not-Fat
T	Testosterone
T-AOC	Total Antioxidant Capacity
TMR	Total Mixed Ration

## References

[1] Guan M.X., Zhong L.W., Wei P.L., Zhang R.Y., Wu L., Panaer, Gong P. (2024). Meat quality analysis of Turpan black sheep. Herbiv. Livest..

[2] Wang J.C., Wang Z.Y., Hou S.Z., Yang C. (2025). Research progress on effects of maternal nutritional intake during lactation on lamb growth and development. Feed. Res..

[3] Yu L., Wang Z.B., Wang Q.H., Kuang H.X. (2018). Research Progress on Pharmacological Effects of Flavonoids in Astragali Radix. Inf. Tradit. Chin. Med..

[4] Miao S.C., Niu X.Y., Xing Y.Y., Li D.B. (2025). Biological functions of plant extracts and their regulatory effects on lactation performance of dairy cows. Chin. J. Anim. Sci..

[5] Xiang Y., Zhu S., Wang R., Huang J., Li H., Dai D., Yang Y., Hu H., Wei Y., Hu T. (2026). Poria cocos polysaccharide alleviates multidrug-resistant *Klebsiella pneumoniae*-induced acute lung injury by modulating the gut–lung axis via gut microbiota remodeling Available to Purchase. Food Funct..

[6] Dong M.Y., Li J.J., Yang D.L., Li M.F., Wei J.H. (2023). Biosynthesis and pharmacological activities of flavonoids, triterpene saponins and polysaccharides derived from Astragalus membranaceus. Molecules.

[7] Deng Y.A., Yang Z.R., Deng W., Cheng J.L. (2018). Pharmacokinetic study on five active ingredients of broken-wall and traditional astragalus decoction pieces in rats. World Sci. Technol.-Mod. Tradit. Chin. Med. Mater. Med..

[8] Zhu X.Y., Xu H.N. (2021). Characteristics of fermented Chinese herbal medicine additives and effect on animal production and economic benefits. Feed. Res..

[9] Lu H., Zhao G.D., Li T.T., Zhou Y., Lu T.T., Wei X.Y., Zhai J.H., Riyimu R., Lv H.B., Wang X.H. (2026). The effects of supplementing Astragalus and fermented Astragalus on lactation performance, rumen microbiota, and lamb weight gain in Turpan black sheep. Front. Microbiol..

[10] Han S.W., Xu G.W., Zhang K., Ahmad S., Wang L., Chen F.B., Liu J.H., Gu X.Y., Li J.X., Zhang J.Y. (2024). Fermented *Astragalus* Powder, a New Potential Feed Additive for Broilers to Improve the Growth Performance and Health. Animals.

[11] (2021). Ministry of Agriculture and Rural Affairs of the People’s Republic of China; Feeding Standard of Meat-Producing Sheep and Goats.

[12] (2014). Determination of Moisture in Feedstuffs.

[13] (2025). Determination of Crude Ash in Feedstuffs.

[14] (2006). Crude Protein in Animal Feed, Combustion Method. Official Methods of Analysis. 18th ed..

[15] (2005). Calcium in Feeds, o-Cresolphthalein Colorimetric Method. Official Methods of Analysis. 18th ed..

[16] (2005). Phosphorus in Feeds, Ammonium Vanadate Colorimetric Method. Official Methods of Analysis. 18th ed..

[17] (2006). Amylase-Treated Neutral Detergent Fiber in Feeds. Official Methods of Analysis. 18th ed..

[18] (2006). Acid Detergent Fiber and Lignin in Animal Feeds. Official Methods of Analysis. 18th ed..

[19] Benson M.E., Henry M.J., Cardellino R.A. (1999). Comparison of weigh-suckle-weigh and machine milking for measuring ewe milk production. J. Anim. Sci..

[20] Zhao M., Wang Y., Liang Y.T., Wang X., Du R.P. (2026). Effects of dextran and polyglutamic acid on ruminal fermentation parameters, microflora and metabolome of periparturient dairy goats. Chin. J. Anim. Nutr..

[21] Li Q.S., Huo J.B., Ni G.F., Zhang F., Zhang S.Z., Zhang X.M., Wang R., Jiao J.Z., Yu Z.T., Pu X.X. (2025). Reductive acetogenesis is a dominant process in the ruminant hindgut. Microbiome.

[22] Liu M.J., Zhang Y.L., Liu Y.C., Li Y.Y., Wang Z.J., Ge G., Jia Y.S., Du S. (2024). Effect of fermented total mixed rations on rumen microbial communities and serum metabolites in lambs. Grassl. Res..

[23] Gao Y.F., Ouyang K.H., Qu M.R., Xiong X.W., Wen Q.Q., Xu L.J. (2016). Analysis of ruminal bacterial diversity of Jinjiang cattle using MiSeq sequencing technology. Chin. J. Anim. Nutr..

[24] Khafipour E., Li S., Tun H.M., Derakhshani H., Moossavi S., Plaizier J.C. (2016). Effects of grain feeding on microbiota in the digestive tract of cattle. Anim. Front..

[25] Ran H., Zhu S.X., Liu T., Xu J.F., Wang J., An L.J., Pan F.M., Zhang H., Wang X.J., Xu G.Y. (2025). Effects of astragalus polysaccharide powder on rumen in vitro fermentation parameters and microflora of Hu sheep. Chin. J. Anim. Nutr..

[26] Šuchová K., Fehér C., Ravn J.L., Bedő S., Biely P., Geijer C. (2022). Cellulose- and xylan-degrading yeasts: Enzymes, applications and biotechnological potential. Biotechnol. Adv..

[27] Sha Y.Z., Liu X., Li X.X., Wang Z.W., Shao P.Y., Jiao T., He Y.Y., Zhao S.G. (2024). Succession of rumen microbiota and metabolites across different reproductive periods in different sheep breeds and their impact on the growth and development of offspring lambs. Microbiome.

[28] Zhang D.L., Chen J.H. (2002). Observation on 35 cases of viral hepatitis with hyperbilirubinemia treated by astragalus injection. J. Pract. Med. Tech..

[29] Zhu Y.H., Chai J. (2021). Effects of Astragalus Qi-tonifying decoction combined with reduced glutathione on liver function, oxidative stress and inflammation in patients after interventional therapy for liver cancer. Shaanxi J. Tradit. Chin. Med..

[30] Zhou W.J., Ji X.T., Zheng L., Yang G.P., Liu T.Z. (2022). Producing high value unsaturated fatty acid by whole-cell catalysis using microalga: A case study with Tribonema minus. Biotechnol. Bioeng..

[31] Kengo T., Toshiaki T., Hirokazu Y., Takahiro S., Rie I., Hirotoshi E., Hidetsugu S., Ryota H., Soichiro M., Toshifumi H. (2011). Plasma free myristic acid proportion is a predictor of nonalcoholic steatohepatitis. Dig. Dis. Sci..

[32] Xu L., Zou X.Y., Gao Z.Q., Mao C.F., Su H., Li C.Y., Chen N. (2021). Improved fatty acid profile reduces body fat and arterial stiffness in obese adolescents upon combinatorial intervention with exercise and dietary restriction. J. Exerc. Sci. Fit..

[33] Moreira V., Brasili E., Fiamoncini J., Marini F., Miccheli A., Daniel H., Lee J.J.H., Hassimotto N.M.A., Lajolo F.M. (2018). Orange juice affects acylcarnitine metabolism in healthy volunteers as revealed by a mass-spectrometry based metabolomics approach. Food Res. Int..

[34] Zhao T.Y., Yang X.F., Liu Z.M., Liu Y.J., Wang C.J., Wei J. (2023). Astragaloside IV and its application prospect in animal husbandry production. China Anim. Health.

[35] Li B.J. (2025). Effects of astragalus polysaccharides on growth performance, immune function and rumen fermentation parameters of heat-stressed beef cattle. China Feed..

[36] Jiang S.S., Li Z.J., Di H.S., Cao Y., Yuan H.G., Zhu J.P. (2026). Effects of astragalus polysaccharides on growth performance, immune function and intestinal flora of puppies. Feed. Res..

[37] Tan C.P., Li F.F., Huang Y.J., Chen H., Yu F.Y. (2024). Effects of dietary astragalus polysaccharides on growth performance, immunity and antioxidant function of beef cattle. China Feed..

[38] Yang Y.W., Li H.J., Zhang K., Ding W., Ma Q. (2023). Study on effects of astragaloside IV on immune function and antioxidant capacity of weaned stressed calves. China Anim. Health.

[39] Wang Y.F. (2020). Study on the Effect and Mechanism of Astragaloside IV on Antioxidant Function of Calves. Master’s Thesis.

[40] Cao T.T., Huang Y.Z., Huang Y.L., Du J., Su S.F., He H., Li L.J., Huang D.F., Zhang J., Hao Y.F. (2026). Effects of fermented astragalus on production performance, serum immune indexes and digestive function of geese. Chin. J. Anim. Sci..

[41] Xu J.Y., Wang R.C. (2026). Extraction and antioxidant activity of total saponins from astragalus residue fermented by mixed strains. China Food Addit..

[42] Lima L.S., Palin M.F., Santos G.T., Benchaar C., Petit H.V. (2015). Effects of supplementation of flax meal and flax oil on mammary gene expression and activity of antioxidant enzymes in mammary tissue, plasma and erythrocytes of dairy cows. Livest. Sci..

[43] Wang Y.C., Wei B.Q., Chen L., Liu Y.X. (2024). Biological functions of astragalus polysaccharides and its application progress in livestock and poultry production. Chin. J. Anim. Nutr..

[44] Yang C., Du M., Ahmad A.A., Cheng Y., Gebeyew K. (2025). Passive immunity establishment through colostral IgG absorption in neonatal ruminants: Foundation for efficient ruminant production. Animals.

[45] Awangcuoji, Wu Y.J., Suolangda, Ba G., Renqingcuomu, De J., Dan Z. (2025). Study on protein requirement of Tibetan cashmere goats in late gestation. Mod. J. Anim. Husb. Vet. Med..

[46] Qi C.X., Yang K.D., Qi Y.Y., Ju H.M. (2016). Research progress on autocrine mechanism of growth hormone. Prog. Vet. Med..

[47] Cai P., Lei Y., Yu W.L., Yang Y., Zhao S.L., Adilai A., Zhao H.Q. (2023). Expression levels of ghrelin and IGF-I; system genes in sheep organs. Heilongjiang Anim. Sci. Vet. Med..

[48] Oksbjerg N., Gondret F., Vestergaard M. (2004). Basic principles of muscle development and growth in meat-producing mammals as affected by the insulin-like growth factor (IGF) system. Domest. Anim. Endocrinol..

